# Storm: Incorporating transient stochastic dynamics to infer the RNA velocity with metabolic labeling information

**DOI:** 10.1371/journal.pcbi.1012606

**Published:** 2024-11-18

**Authors:** Qiangwei Peng, Xiaojie Qiu, Tiejun Li

**Affiliations:** 1 LMAM and School of Mathematical Sciences, Peking University, Beijing, China; 2 Department of Genetics, Stanford University School of Medicine, Stanford, California, United States of America; 3 Basic Sciences and Engineering Initiative, Betty Irene Moore Children’s Heart Center, Lucile Packard Children’s Hospital, Stanford, California, United States of America; 4 Department of Computer Science, Stanford University, Stanford, California, United States of America; 5 Stanford Cardiovascular Institute, Stanford University, Stanford, California, United States of America; 6 Center for Machine Learning Research, Peking University, Beijing, China; Max-Delbruck-Centrum fur Molekulare Medizin in der Helmholtz-Gemeinschaft, GERMANY

## Abstract

The time-resolved scRNA-seq (tscRNA-seq) provides the possibility to infer physically meaningful kinetic parameters, e.g., the transcription, splicing or RNA degradation rate constants with correct magnitudes, and RNA velocities by incorporating temporal information. Previous approaches utilizing the deterministic dynamics and steady-state assumption on gene expression states are insufficient to achieve favorable results for the data involving transient process. We present a dynamical approach, Storm (Stochastic models of RNA metabolic-labeling), to overcome these limitations by solving stochastic differential equations of gene expression dynamics. The derivation reveals that the new mRNA sequencing data obeys different types of cell-specific Poisson distributions when jointly considering both biological and cell-specific technical noise. Storm deals with measured counts data directly and extends the RNA velocity methodology based on metabolic labeling scRNA-seq data to transient stochastic systems. Furthermore, we relax the constant parameter assumption over genes/cells to obtain gene-cell-specific transcription/splicing rates and gene-specific degradation rates, thus revealing time-dependent and cell-state-specific transcriptional regulations. Storm will facilitate the study of the statistical properties of tscRNA-seq data, eventually advancing our understanding of the dynamic transcription regulation during development and disease.

## Author summary

Intricate regulation of RNA biogenesis, such as RNA transcription, splicing and degradation, plays a critical role in most biological processes. Previous approaches have leveraged a deterministic model of spliced and unspliced RNAs to estimate kinetic parameters and quantify RNA velocity, the rate of changes in gene expression states in single cells. However, accurately estimating meaningful kinetic parameters and RNA velocity is hindered by biased capture of unspliced RNA and absence of temporal information in conventional scRNA-seq. Significant advances have been made in better measuring RNA kinetics with metabolic labeling enabled scRNA-seq, however, computational tools to analyze them lag far behind. Prior work of Dynamo provides one of the early solutions to properly model RNA metabolic labeling data, but its method still largely uses a deterministic model that only utilizes part of extreme cells and is unable to analyze datasets with significant transient dynamics. To address these challenges, we developed Storm that explicitly models transient stochastic RNA dynamics. Importantly, Storm models RNA kinetics with stochastic differential equations that explicitly account for biological and cell-specific noises. Storm is generally applicable to many metabolic labeling scRNA-seq datasets and we demonstrate excellent performance of Storm in fitting the data to capture the transient dynamics under various noise models.

## Introduction

Cells are dynamic identities that are subject to intricate transcriptional and post-transcriptional regulations. Understanding the tight regulation of the RNA life cycle will shed light on not only the regulatory mechanism of RNA biogenesis, but also cell fate transitions. Based on the observation that most scRNA-seq approaches capture both premature unspliced mRNA and mature spliced mRNA information, La Manno et al. [1] pioneered the concept of RNA velocity or the time derivative of spliced RNA to reveal the local fate of each individual and designed an RNA kinetic parameter inference method called velocyto based on the steady state assumption. In a later work, scVelo [2] relaxed the steady-state assumption and proposed a dynamic RNA velocity model to infer gene-specific reaction rates of transcription, splicing and degradation as well as cell-specific hidden time using the expectation-maximization (EM) algorithm. Li et al. [3, 4] derived a stochastic model of RNA velocity based on the chemical master equation (CME) satisfied by the probability mass function (PMF) rather than the deterministic ordinary differential equation (ODE) satisfied by the mean, and presented a mathematical analysis framework of RNA velocity. More general studies on the analytical solution of the CME for monomolecular reaction systems are also discussed in [5]. In addition, a rigorous and detailed analysis of the entire workflow for RNA velocity is also provided in [6]. MultiVelo [7] extends the dynamic RNA velocity model by incorporating epigenome data that can be jointly measured with emerging multi-omics approaches. Protaccel [8] extends the concept of RNA velocity to protein. UniTVelo [9] uses a top-down design for more flexible estimation of the RNA velocity, as opposed to the usual bottom-up strategy. DeepVelo [10] uses graph convolutional neural networks to infer cell-specific parameters to extend RNA velocity to cell populations containing time-dependent dynamics and multiple lineages which were proven to be challenging in previous methods [11]. Other deep learning-based approaches include VeloAE [12], VeloVI [13], VeloVAE [14], LatentVelo [15], cellDancer [16], and so on. However, due to the absence of physical time information, the above methods usually suffer the issue of scale invariance, that is, amplifying the parameters by an arbitrary amount will not change the solution if the time shrinks with the same amount, e.g., the exact physical time remains undetermined. This issue makes the inferred parameters and the RNA velocity have physical significance only up to a multiplicative constant [3]. In addition, the missing time information enters the model as hidden variables, which makes the parameter inference difficult.

Technological innovations in scRNA-seq now enable us to directly measure the amount of newly synthesized mRNA molecules over a short period of time, either through chemically introduced mutations in the sequencing reads or direct biotin pull-down of RNA analogs such as 4sU metabolically labeled RNA molecules, which subtly introduces physical time information. These time-resolved metabolic labeling–augmented scRNA-seq (tscRNA-seq) include scSLAM-seq [17], scNT-seq [18], sci-fate [19], NASC-seq [20], scEU-seq [21], PerturbSci-Kinetics [22] and others [23–25]. Qiu et al. [26] recently developed Dynamo to reconstruct analytical vector fields from discrete RNA velocity vectors by taking advantage of tscRNA-seq data to infer more robust and time-resolved RNA velocity, however, they only used the deterministic model and largely relied on the steady-state assumption. CellRank2 [27] also focuses on pulse and chase tscRNA-seq data, but ignores the broader one-shot tscRNA-seq data, and relies on deterministic models for parameter inference.

To overcome the shortcomings of Dynamo and fully explore the potential of tscRNA-seq data, we present the Storm approach (Stochastic models of RNA metabolic-labeling) to improve the estimation of RNA kinetic parameters and the inference of the RNA velocity of the metabolic labeling scRNA-seq data by incorporating the transient stochastic dynamics of gene expressions. Importantly, we focus on modeling the kinetics/pulse metabolic labeling data as it follows the RNA synthesis across multiple short time periods and is thus ideal for capturing temporal RNA kinetics. In order to properly model both biological noise and cell-specific technical noise (due to the variations in sequencing depth across individual cells and dropout resulting from imperfect RNA capture in scRNA-seq), we implemented in Storm three stochastic models of new mRNA (or new unspliced and spliced mRNA). Depending on the biological processes considered, Storm indicates that new mRNA sequencing data obeys different types of cell-specific Poisson (CSP) distributions. On this basis, Storm also includes hypothesis testing, parameter inference and goodness of fit evaluation methods for CSP-type distribution. In addition, we analyze the similarities and differences of the model considering RNA splicing or not. For one-shot data containing both unspliced unlabeled (uu), unspliced labeled (ul), spliced unlabeled (su) and spliced labeled (sl) RNA, we devise a two-stage parameter inference method that does not rely on steady-state assumption to infer the absolute magnitude of the kinetic parameters. For one-shot data containing only new RNA and total RNA, we introduce the steady-state assumption to make the parameter inference possible. We verified the effectiveness of Storm in the cell cycle data set of kinetic experiments from the scEU-seq study [21] and several one-shot datasets, including scSLAM-seq, scNT-seq, sci-fate and PerturbSci-Kinetics. Storm is incorporated in Dynamo [26] of the Aristotle ecosystem that facilitates rich downstream analytical vector field modeling.

## Results

### Overall description of Storm

**Fig 1:**
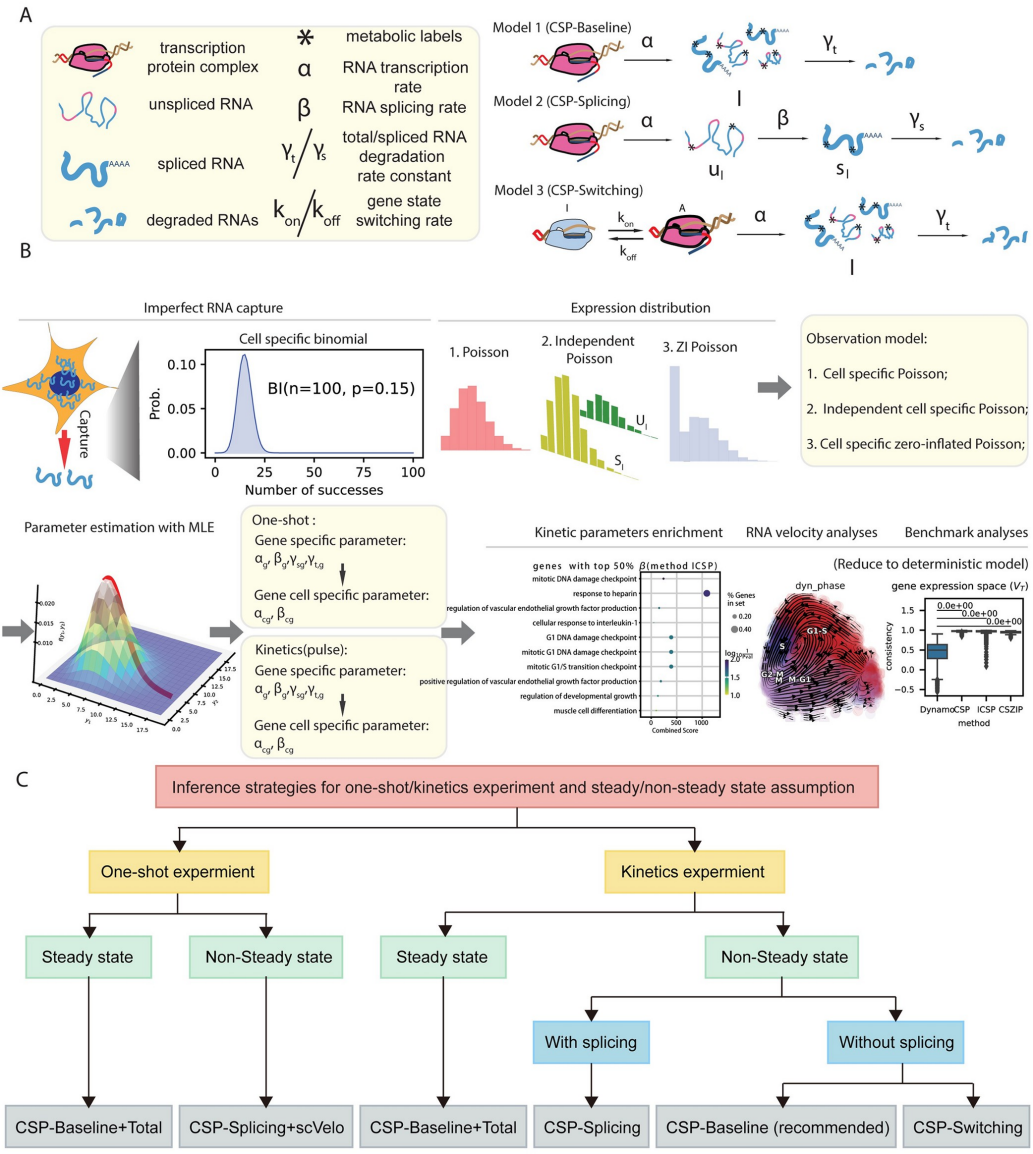
Schematic overview of Storm. **A.** Three models of RNA life cycle considering different biological processes: **Model 1 (CSP-Baseline)**: Reaction dynamics model for new RNA *l*(*t*) ignoring the splicing process, where *α* is the transcription rate and *γ*_*t*_ is the total mRNA degradation rate. **Model 2 (CSP-Splicing)**: Reaction dynamics model of new unspliced and spliced mRNA (*u*_*l*_(*t*), *s*_*l*_(*t*)) considering the splicing process, where *β* is the splicing rate, *γ*_*s*_ is the spliced mRNA degradation rate, and *α* is the same as **Model 1**. **Model 3 (CSP-Switching)**: Reaction dynamics model of new RNA *l*(*t*) considering gene state switching, where *α* and *γ*_*t*_ are the same as in **Model 1**, *k*_on_ is the rate at which the gene switches from the inactive state to the active state, *k*_off_ is the opposite. **B.** Complete workflow diagram for parameter inference and downstream analysis based on stochastic dynamics of new mRNA considering technical noise. **C.** Specific parameter inference strategies for one-shot/pulse experiments and steady-state/non-steady-state assumption.

We established three stochastic gene expression models for new mRNA (or new unspliced and spliced mRNA) (Fig 1A) for the inference of the RNA kinetic parameters and thus the RNA velocity in the Storm approach. In Model 1, only transcription and mRNA degradation were considered. In Model 2, we considered transcription, splicing, and spliced mRNA degradation. And in Model 3, we considered the switching of gene expression states, transcription in the active state, and mRNA degradation.

The complete workflow of Storm is demonstrated in Fig 1B. We first analytically solve the new RNA (or new unspliced and spliced mRNA) stochastic dynamics corresponding to the above three models, which are Poisson distribution, independent Poisson distribution and zero-inflated Poisson distribution, respectively. In addition, we model the technical noise as the cell-specific binomial distribution. By integrating the biological noise and the technical noise together, we obtain the distribution for the measured number of new/labeled mRNA molecules (or new unspliced and spliced mRNA molecules), which are cell-specific Poisson distribution, independent cell-specific Poisson distribution and cell-specific zero-inflated Poisson distribution, respectively. Therefore, we call the three models CSP-Baseline, CSP-Splicing, and CSP-Switching for easy memory and use them later to better distinguish these three models. Maximum likelihood estimation (MLE) is used to fit the data and make inferences for the parameters shown in the corresponding models.

**Fig 2:**
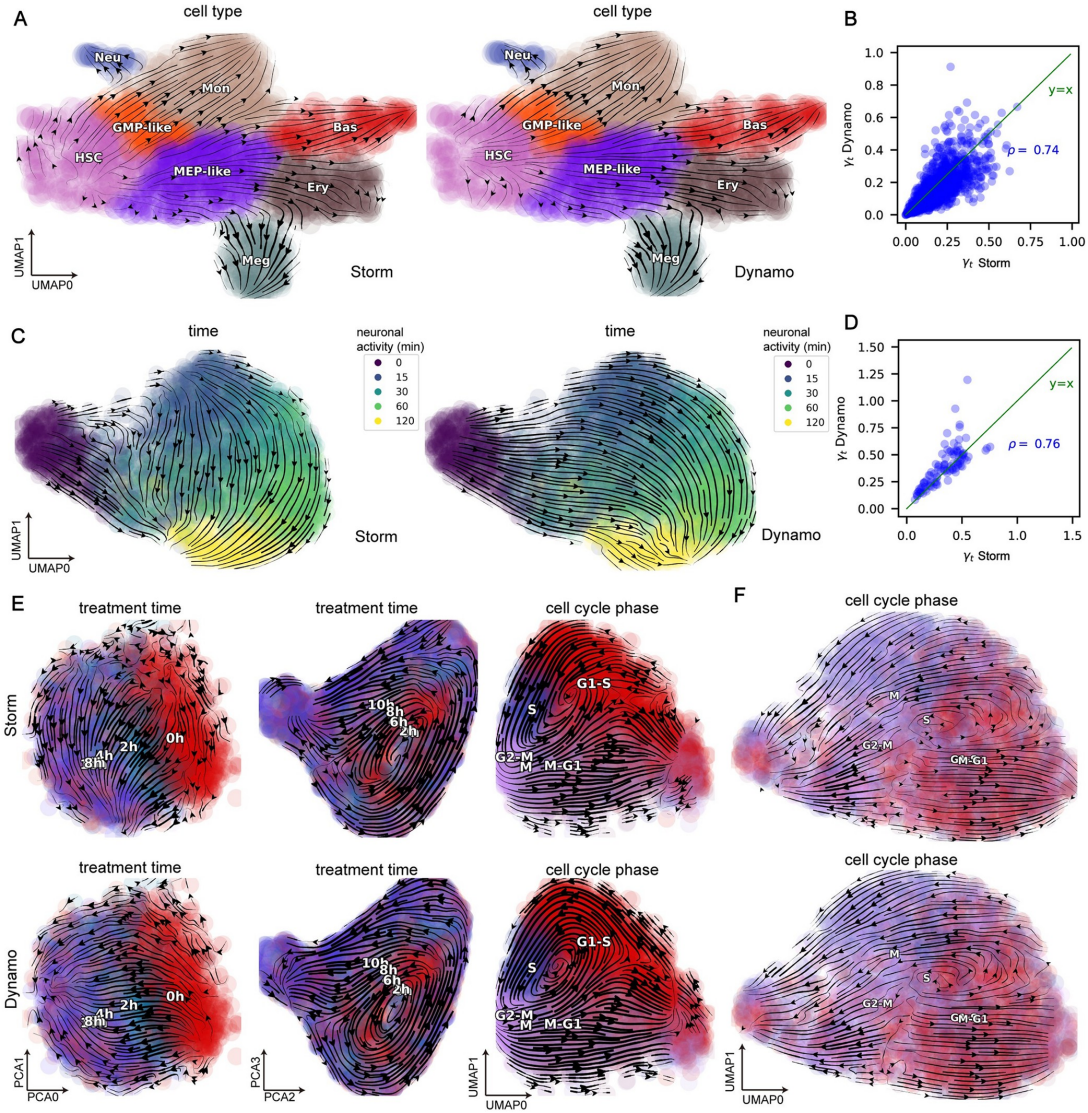
Stochastic model combined with steady-state assumptions for one-shot data without splicing information. **Storm in this figure refers to the inference strategy of CSP-Baseline model combined with the steady state assumption**. **A.** Streamline projected in the UMAP space plots of primary human HSPCs datasets from scNT-seq [26]. **B.** Degradation rates *γ*_*t*_ estimated with steady-based method in Storm compared to that of the Dynamo method in the primary human HSPCs datasets. **C.** Streamline projected in the UMAP space plots of neuronal activity under KCl polarization datasets from scNT-seq [18]. **D.** Same as **B.**, but for the neuronal activity datasets. **E.** Streamline plots of the sci-fate dataset [19] reveal two orthogonal processes of GR response and cell-cycle progression. From left to right: streamline plot on the first two PCs, the second two PCs, and the first two UMAP components that are reduced from the four PCs, respectively. The first row is the result of Storm and the second row is the result of Dynamo. **F.** Streamline projected in the UMAP space plots of the dataset from PerturbSci-Kinetics [22].

To ensure the general applicability of Storm in common nascent RNA labeling schemes, such as one-shot or kinetics/pulse experiments (See Figure 2 of Qiu, et. al [26]), we designed specific estimation strategies for each labeling scheme(Fig 1C). For the one-shot labeling experiments with only new RNA and total RNA data, since there is only one labeling duration and the lack of splicing data, the steady-state assumption under the stochastic dynamics framework is reinvoked to infer parameters (Fig 1C Left). For the one-shot labeling experiments with uu, ul, su and sl RNA, we design a two-stage approach that does not rely on the steady-state assumption. More specifically, we first use scVelo [2] to determine the relative size of the kinetic parameters, and then use CSP-Splicing to determine the absolute size (Fig 1C Left). For kinetics/pulse-labeling experiments with multiple labeling durations, the transient stochastic dynamics framework is used without the steady-state assumption (Fig 1C Right). CSP-Baseline is recommended if the concern is RNA velocity, and CSP-Splicing should be used if the concern also includes splicing dynamics. Although steady-state assumption can also be included, we recommend that non-steady-state approach should be used unless the user has sufficient knowledge of the biological process being studied. Furthermore, the goodness-of-fit index based on deviance commonly used in generalized linear models is utilized to quantify the goodness of fit of our models in kinetics/pulse datasets. The index is then used to select genes that are more consistent with model assumptions for later downstream analysis, such as the enrichment analysis of different gene-specific parameters. Furthermore, we relaxed the previous assumption of constant parameters in genes or cells and assumed that only degradation rates (*γ*_*t*_ in CSP-Baseline and CSP-Switching; *γ*_*s*_ in CSP-Splicing) are constant while the other parameters are cell-specific and depend on the state of gene expression in each cell. This relaxation would be useful for modeling lineage-specific kinetics resulted from hierarchical lineage bifurcation, which is common in cell development. Finally, in order to calculate and visualize the RNA velocity, we reduced the considered stochastic models to derive the deterministic equation for the mean gene expression. The inferred parameters, after filtering with the goodness-of-fit index are then used in RNA velocity analysis and visualization. Notably, to demonstrate Storm’s performance, we conducted systematic comparison with the state-of-the-art method Dynamo [26] for processing metabolic labeling scRNA-seq experiment datasets.

In the continued subsections we will present the details of each step in the Storm workflow, starting from the introduction of our mathematical models.

### CSP modeling of counts data with metabolic labeling information

We proposed and analytically solved three aforementioned stochastic gene expression models for the dynamics of new mRNAs (or new unspliced and spliced mRNAs).

For simplicity of modeling, we followed [1, 2] to assume that the genes are independent. In the stochastic gene expression model, the generation of new/labeled mRNA $\tilde{l}\left(t\right)$ (or new unspliced and spliced mRNA $\left({\tilde{u}}_{l}\left(t\right),{\tilde{s}}_{l}\left(t\right)\right)$) is a stochastic process, and we are interested in the evolution of its PMF over time, which is denoted by

$$\begin{array}{c} \begin{array}{c} {\tilde{P}}_{n}\left(t\right)≔\text{Prob}(\tilde{l}\left(t\right)=n),\;n\in N\\ {\tilde{P}}_{mn}\left(t\right)≔\text{Prob}(({\tilde{u}}_{l}\left(t\right),{\tilde{s}}_{l}\left(t\right))=\left(m,n\right)),\;\left(m,n\right)\in N^{2}. \end{array} \end{array}$$ (1)

In CSP-Baseline and CSP-Splicing, Since the initial value of $\tilde{l}\left(t\right)$ (or $\left({\tilde{u}}_{l}\left(t\right),{\tilde{s}}_{l}\left(t\right)\right)$) is 0, we obtained the following closed-form solution (see “Methods” section).

$$\begin{array}{c} \begin{array}{cc} \text{CSP-Baseline:}\; & {\tilde{P}}_{n}\left(t\right)\;\;=\frac{a^{n}\left(t\right)}{n!}e^{-a(t)},\;n\in N,\\ \text{CSP-Splicing:}\; & {\tilde{P}}_{mn}\left(t\right)=\frac{b^{m}\left(t\right)c^{n}\left(t\right)}{m!n!}e^{-b(t)-c(t)},\;\left(m,n\right)\in N^{2}, \end{array} \end{array}$$ (2)

where *a*(*t*), *b*(*t*) and *c*(*t*) are solutions to the corresponding deterministic equation, which means that $\tilde{l}\left(t\right)$ obeys the Poisson distribution with mean *a*(*t*) in CSP-Baseline, and $\left({\tilde{u}}_{l}\left(t\right),{\tilde{s}}_{l}\left(t\right)\right)$ obey independent Poisson distributions with mean *b*(*t*) and *c*(*t*) in CSP-Splicing. Here *α*, *β* are the transcription and splicing rates, and *γ*_*s*_, *γ*_*t*_ are the spliced and total mRNA degradation rates, respectively. CSP-Switching can be solved similarly analytically under the assumption that the switching rates are much smaller than the transcription and degradation rates (see “Methods” section).

We also specifically modeled technical noise of the measured number of new RNA (or new unspliced and spliced mRNA) molecules in scRNA-seq experiments. Such noises often lead to dropout of RNA measurements during the sequencing process and generally result in variations in sequencing depth across cells. To account for the noise, in Storm we modeled the dropout process of sequencing technology as cell-specific binomial distributions. Adopting the common practice in many preprocessing pipelines through a size factor to normalize the data [1, 2, 10, 13, 26], we assumed that the total numbers of mRNA molecules across all genes in different cells are close. Probabilistically, this assumption implies that

$$p_{j}\propto n_{j},$$

where *p*_*j*_ is the probability of mRNA molecules being captured in cell *j* and *n*_*j*_ is the total number of mRNA molecules across all genes in cell *j* in scRNA-seq experiments.

Combining the stochastic models for biological and technical noise, we can obtain different formalisms of the distribution for the measured number of new/labeled mRNA molecules *l*(*t*) (or new unspliced and spliced mRNA molecules (*u*_*l*_(*t*), *s*_*l*_(*t*))) in the scRNA-seq experiments (see “Methods” section) for each model. Specifically, in CSP-Baseline, *l*(*t*) obeys the cell-specific Poisson (CSP) distribution, that is,

$$\begin{array}{c} P_{n,j}\left(t\right)=\frac{{\left(p_{j}a\left(t\right)\right)}^{n}}{n!}e^{-p_{j}a\left(t\right)}, \end{array}$$ (3)

where *P*_*n*,*j*_(*t*) is the PMF for the measured number of new mRNA molecules in cell *j*. In CSP-Splicing, (*u*_*l*_(*t*), *s*_*l*_(*t*)) obeys the independent cell-specific Poisson (ICSP) distribution, that is,

$$\begin{array}{c} P_{mn,j}\left(t\right)=\frac{{\left(p_{j}b\left(t\right)\right)}^{m}}{m!}e^{-p_{j}b\left(t\right)}\frac{{\left(p_{j}c\left(t\right)\right)}^{n}}{n!}e^{-p_{j}c\left(t\right)}, \end{array}$$ (4)

where *P*_*mn*,*j*_(*t*) is the joint PMF for the measured number of new unspliced and spliced mRNA molecules in cell *j*. The derivation of CSP-Switching is similar (see “Methods” section). We call the above distributions as *cell-specific* because different cells obey the distributions with different parameters. Finally for labeling efficiency, we did not model it directly but followed Dynamo using GRAND-SLAM [33] to correct the new RNA data in advance.

Note that Grün et al. also modeled the scRNA-seq data by integrating biological noise and technical noise [34]. Our work is different from them in the following aspects: (1) Our work models the transient dynamics of new mRNA and solves their distribution for the proposed stochastic models analytically. However, in [34], they instead modeled the total mRNA and derived that the biological noise follows a negative binomial distribution as the steady state of the transcriptional bursting model. (2) Our work accurately models the technical noise as a cell-specific binomial distribution, while they approximated the cell-specific binomial distribution with a Poisson distribution and modeled the capture probability as a random variable subject to the Gamma distribution, which finally leads to a negative binomial distribution (Poisson-Gamma mixture distribution) of the technical noise.

As one-shot labeling experiments are much more convenient than pulse experiments in practice, in the following, we will first demonstrate how Storm can be applied to the one-shot case. We will then extensively show Storm’s power in analyzing the pulse datasets.

### Stochastic models combined with steady-state assumptions for one-shot data without splicing information

For one-shot data without splicing information, we designed the corresponding parameter inference method which invokes the steady-state assumption under the stochastic model, focusing specifically on CSP-Baseline (see “Methods” section). Similar steady-state methods of the stochastic model can also be designed for both CSP-Splicing and CSP-Switching as well, although they are not the focus of this paper.

We validated our method in several one-shot datasets (Fig 2 and S1 Fig). We first analyzed a primary human HSPCs datasets from scNT-seq [18]. Both Dynamo and Storm reveal a smooth transition of HSCs into MEP-like and GMP-like cells, which further ramify into Meg/Ery/Bas lineages and Mon/Neu lineages, respectively, which is consistent with the established knowledge of hematopoiesis (Fig 2A). Next, we analyzed the neuronal activity dataset from the scNT-seq study [18] to investigate cellular polarization dynamics after KCl treatment. Dynamo and Storm both revealed a coherent transition that nicely follows the temporal progression from time point 0 to 15, 30, 60 and finally 120 minutes (Fig 2C). We also analyzed a dataset from the sci-fate study [19] in which cell cycle progression and glucocorticoid receptor (GR) activation were explored. Similar to Dynamo, the RNA velocity flow from our method also revealed a sequential transition of cells following the DEX (dexamethasone) treatment times in the first two principal components (PCs) (Fig 2E Left). In the second two PCs, we observed an orthogonal circular progression of the cell cycle (Fig 2E Middle). From the first two UMAP dimensions projected further from the four PCs, we observed a combined dynamics of GR responses and cell cycle progression (Fig 2E Right). We analyzed a dataset from PerturbSci-Kinetics [22], Dynamo and Storm observed similar results (Fig 2F). Additionally we analyzed mouse fibroblast cells dataset from scSLAM-seq [17]. We observed that Dynamo and Storm inferred similar velocities, and they both further discriminated infected from non-infected cells (S1A Fig).

Finally, we quantitatively compared the degradation rates *γ*_*t*_ inferred by the two methods (Fig 2B and 2C and S1B and S1D Fig). The inferred results of the two methods are highly correlated, with values of 0.74, 0.76, 0.80, 0.91, and 0.87 respectively on these five datasets. The absolute differences are also low, and the inferred results are mostly distributed around the green line *y* = *x*. We think that the possible reason for this is that the steady-state assumption plays a decisive role. Such methods may fail when the steady-state assumption is violated, so it is important to design methods for one-shot experiments that do not rely on the steady state assumption.

### Storm analyzes one-shot data with both splicing and labeling information without steady-state assumption

For one-shot data containing both splicing and labeling information, we designed a two-stage parameter inference method that does not depend on the steady-state assumption by first modeling unspliced and spliced RNAs with the dynamic model in scVelo [2] and then unspliced labeled and spliced labeled RNA counts with CSP-Splicing (see “Methods” section). Also distinguishing from scVelo [2] in which velocity genes are picked before running the algorithm, we compute goodness of fit *R*^2^ to pick well-fitting genes in scVelo [2] for downstream analysis (see “Methods” section).

**Fig 3:**
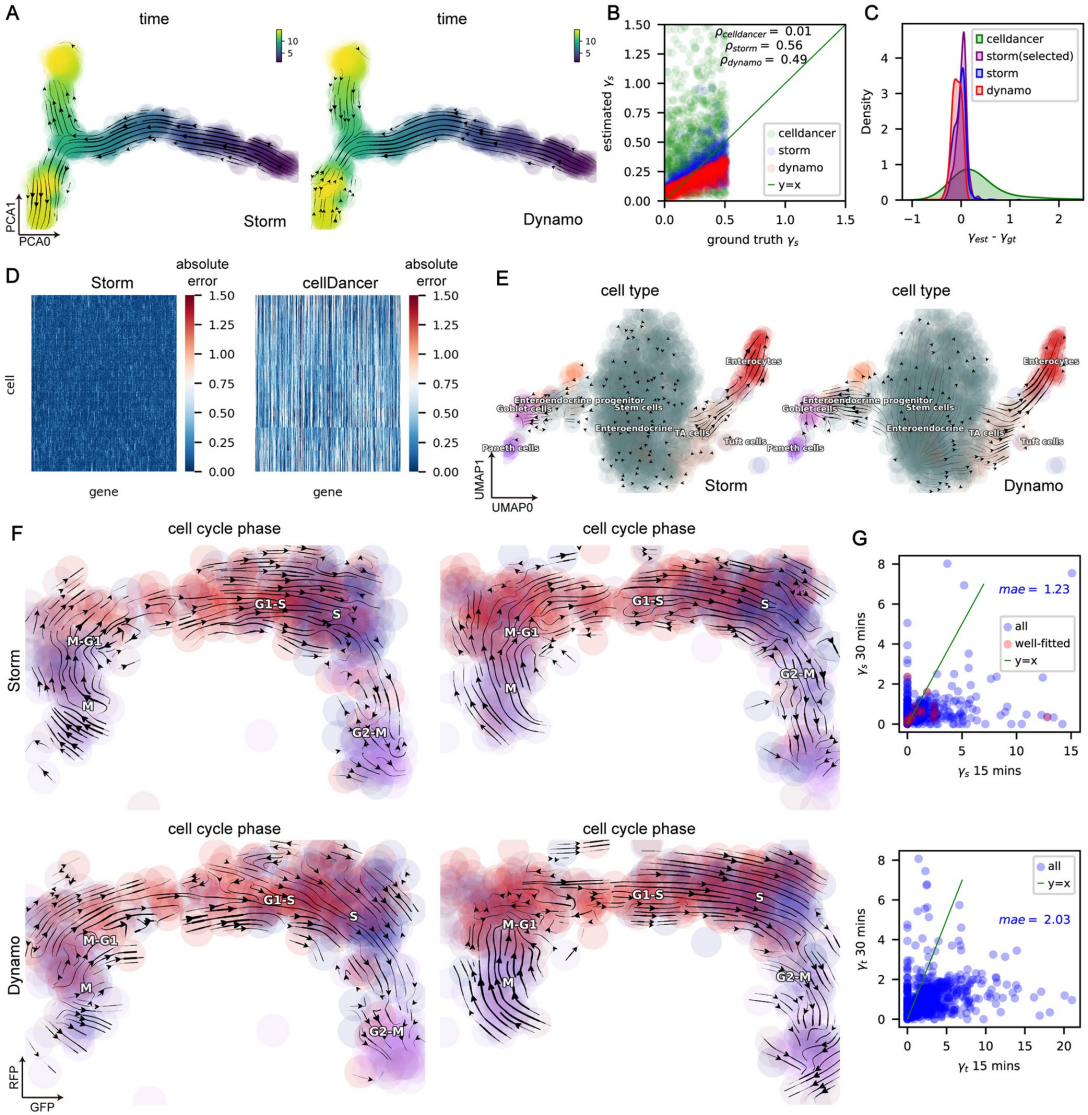
Storm analyzes one-shot data with both splicing and labeling information without steady-state assumption. **Storm in this figure refers to the inference strategy of CSP-Splicing model combined with scVelo**. **A.** Streamline projected in the PCA space plots of one-shot bifurcation simulation data. Left: Storm; Right: Dynamo. **B.** Comparison of the estimated degradation rate with the true degradation rate in one-shot bifurcation simulation data. cellDancer uses the average of cell-wise degradation rates. **C.** Distribution plot of the difference between the estimated degradation rate and the true value, including Storm, Storm (selected) and Dynamo. **D.** Heat map of absolute error between estimated and true gene-cell-wise transcription rates *α* of one-shot bifurcation simulation data. Left: Storm; Right: cellDancer. **E.** Streamline plot in the UMAP space of the murine intestinal organoid system dataset from scEU-seq [21]. **F.** Streamline projected in the RFP_GFP space plots of cell cycle dataset from scEU-seq [21]. On the left is the result of taking only the data labelled with 15 minutes, and on the right is the data labelled with 30 minutes. **G.** Comparison of degradation rates (*γ*_*s*_ in Storm and *γ*_*t*_ in Dynamo) in cell cycle datasets with labeling duration of 15 and 30 minutes.

We validate our method on both simulation and real single cell datasets (Fig 3 and S2 Fig). We first constructed a bifurcated one-shot simulation dataset by following the methods for constructing bifurcated data in SymSim [30] and VeloSim [31] (see “Methods” section). The correct direction of the streamlines started from the right side of the cells on the PCA embedding and then bifurcated into two branches in the middle. We compare the performance of Storm, Dynamo, and a deep learning-based method cellDancer [16] on this simulated dataset, and the results show that only Storm and cellDancer got the correct streamlines (Fig 3A and S2A Fig). In addition we compared the degradation rate values estimated by the different methods with the true values (Fig 3B and 3C). The results show that cellDancer’s estimated values are not in the same order of magnitude as the true values and have a poor correlation (Fig 3B and 3C). This is a difficulty inherent in methods that are missing physical time information. Dynamo and Storm’s estimates are in the same order of magnitude as the true values, but Storm has a lower absolute error and a higher correlation with the true values compared to Dynamo (Fig 3B and 3C). It is also worth noting that the absolute error of the selected genes in Storm is further reduced (Fig 3C), which indicates that the selection strategy is effective. We also compared gene-cell-wise transcription rates. The results show lower absolute errors in estimated transcription rates for Storm and Dynamo (Fig 3D Left and S2C Fig), but much higher for cellDancer (Fig 3D Right). We analyzed the murine intestinal organoid system dataset from scEU-seq [21]. Both Storm, Dynamo and cellDancer observed a bifurcation (Fig 3E and S2B Fig) from intestinal stem cells into the secretory lineage (left) and the enterocyte lineage (right).

To demonstrate the precision and robustness of the Storm method in estimating the one-shot dataset, we benchmarked the estimated kinetic parameters of different subsets of the cell cycle pulse-labeling dataset [21], each with a different duration of labeling. On the 15-minute labeling sub-dataset, Storm recovers a transition that matches well with the cell-cycle progression, while the transition recovered by Dynamo is problematic near the M phase (Fig 3F Left). On the 30-minute labeling sub-dataset, both methods recover the cell cycle progression correctly, but the streamlines of our method are considerably smoother compared to those of Dynamo (Fig 3F Right). In addition, we compared the consistency of degradation rates (*γ*_*s*_ in Storm and *γ*_*t*_ in Dynamo) inferred by the two methods between two sub-datasets with different labeling durations (Fig 3G). The results show that they are similar in terms of correlation, but our method is much smaller in terms of mean absolute error. Notably, although Storm shows higher consistency than Dynamo, it is still not satisfactory, perhaps due to the experimental noises from different labeling durations. Therefore, it is crucial to integrate data of different durations of labeling when a kinetic experiment is available. Furthermore, it is equally important to design methods that do not rely on the steady-state assumption and integrate data of different durations for parameter inference.

### Statistical analysis of cell cycle dataset based on Storm’s stochastic model

Next, we first performed a goodness-of-fit test of the stochastic model proposed in Storm to a cell cycle dataset from scEU-seq [21] with multiple labeling time points to validate our proposals.

When the fixed labeling duration is *t*_fixed_, *a*(*t*_fixed_), *b*(*t*_fixed_) and *c*(*t*_fixed_) are all fixed constants. We can test whether the number of new mRNA molecules in tscRNA-seq within a fixed labeling duration matches the distribution obtained based on the stochastic models (Eqs (3), (4) and (16)), respectively. A common method of testing whether a dataset obeys a given distribution is the chi-square (*χ*^2^) goodness-of-fit test [35]. However, the usual *χ*^2^ test is not directly applicable because in our case different cells obey different distributions with different parameters. By inspecting the mathematical analysis procedure of the *χ*^2^ test [36], we constructed a new asymptotic *χ*^2^ statistics and proposed a modified *χ*^2^ test for our cell-specific distributions (see “Methods” section).

We used the proposed cell-specific *χ*^2^ test in the cell cycle dataset from the scEU-seq study [21], in which cells were labeled for 15, 30, 45, 60, 120 or 180 minutes. Because the labeled unspliced mRNA counts *u*_*l*_(*t*) were too small to be grouped/binned to create a distribution, hypothesis tests were performed only for CSP and CSZIP distributions and not for ICSP distribution. The results are shown in Table 1. We found that some genes were not well determined (especially for cases with a short duration of labeling) in the sense that these genes had too few new mRNA molecules in the tscRNA-seq experiments, which resulted in very few groupings and perfect fittings. With so few mRNA counts for these genes, we were unable to determine whether they obeyed our proposed distribution or not. Moreover, our results revealed that the CSZIP distribution exhibited a better fit with the data than the CSP distribution when focusing on a fixed time point alone, suggesting that the data are indeed zero-inflated.

**Table 1:** The proposed sample-specific hypothesis test results on whether the number of new mRNA molecules in the Cell Cycle dataset obeys the CSP and CSZIP distributions. UTD means that it is unable to determine because there are too few groupings resulting in zero degrees of freedom, when it is always a perfect fit. The significance level is 0.05.

Labeling duration		15mins	30mins	45mins	60mins	120mins	180mins
CSP	Accept	0.116	0.067	0.049	0.062	0.064	0.065
	Reject	0.278	0.568	0.655	0.652	0.695	0.725
	UTD	0.606	0.365	0.296	0.286	0.241	0.210
CSZIP	Accept	**0.351**	**0.467**	**0.472**	**0.476**	**0.459**	**0.459**
	Reject	0.055	0.189	0.266	0.274	0.327	0.344
	UTD	0.594	0.344	0.262	0.250	0.214	0.197

**Fig 4:**
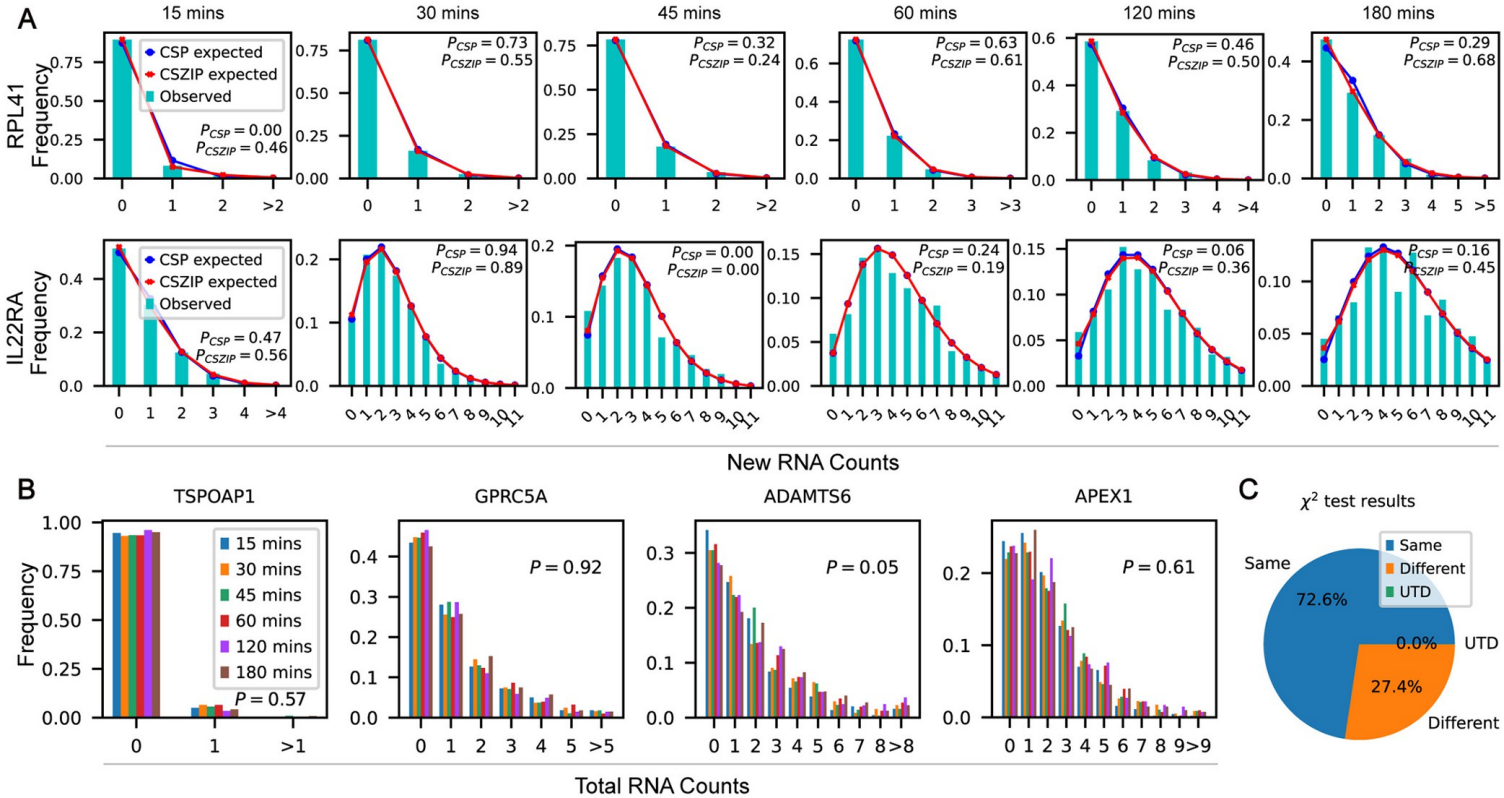
Statistical analysis of cell cycle dataset. **A.** Observed counts, expected counts of CSP distribution, and expected counts of CSZIP distribution of new mRNA molecules of the two example genes *RPL41* and *IL22RA1*. The first row: Fitting results of the *RPL41* gene with a small number of mRNA molecules; The second row: Fitting results of the *IL22RA1* gene with a higher number of new mRNA molecules (truncated to 11 for better visualization). *P*_*CSP*_ and *P*_*CSZIP*_ refer to the p-values of the cell-specific chi-square tests with the corresponding distributions. **B.** Comparison of the total mRNA counts with different labeling durations of the four example genes *TSPOAP1*, *GPRC5A*, *ADAMTS6* and *APEX1*. *P* refers to the p-value of the Chi-square contingency table independence test. **C.** Results of chi-square independence test for total RNA counts (significance level 0.05). “Same” here means accepting the null hypothesis of the chi-square independence test that total RNA counts with different time durations obey the same distribution. “Different” means the opposite.

We next showed the high goodness-of-fit of the CSP and CSZIP distributions on two characteristic genes, namely *RPL41* and *IL22RA1* with an overall low and high gene expression respectively (Fig 4A). Qualitatively, we found that the expected counts of both the CSP and CSZIP distributions matched well with the observed counts for the gene *RPL41*. Quantitatively, the results of the cell-specific chi-square test also showed that the CSP or CSZIP distribution was well satisfies in most labeling durations (Fig 4A first row). Similar results were observed for the gene *IL22RA1* with significantly higher expression (Fig 4A second row). Therefore, we demonstrated CSP and CSZIP distributions accurately describe these two genes and is thus suitable for modeling the tscRNA-seq datasets.

Finally, we found that, for most genes, the number of total mRNA molecules shares the same distribution across different labeling durations. In Fig 4B, we showed the number of total mRNA molecules of four example genes *TSPOAP1*, *GPRC5A*, *ADAMTS6* and *APEX1* is nearly identical across different labeling durations. Quantitatively, we performed a global chi-square independence test on the number of total mRNAs (as distinct from the new mRNAs) with different durations of labeling in all genes and found that, interestingly, there are 72.3% of the genes passed the test at a significance level of 0.05 (Fig 4C). This indicates that a considerable proportion of the number of genes’ total mRNA molecules obeyed the same distribution, consistent with what we observed for the four example genes.

### Storm accurately infers kinetic parameters that leads to rich insights of cell cycle via enrichment analysis

In the kinetic experiments, we relied on three stochastic models without the steady-state assumption to infer different set of kinetic parameters using maximum likelihood estimation (see “Methods” section), namely *α* and *γ*_*t*_ for CSP-Baseline, *α*, *β* and *γ*_*s*_ for CSP-Splicing, and *α*, *γ*_*t*_ and *p*_off_ for CSP-Switching. In addition, we defined the goodness-of-fit of each of the three models (see “Methods” section). According to the goodness-of-fit index, we selected genes that were more consistent with the model assumptions for downstream tasks, such as the enrichment analysis and RNA velocity analysis, etc.

Compared with Dynamo [26], the state-of-the-art method for processing tscRNA-seq datasets, our advantages are mainly in the following aspects: (1) Our method does not require steady-state assumptions on the kinetics experiments while Dynamo heavily relies on the steady-state assumptions; (2) Our stochastic model-based approach is more consistent with real biological process, while Dynamo only utilizes the deterministic model of mean value; (3) Our model takes into account all cells in the inference, while the approach based on steady-state assumptions in Dynamo only considers a small number of cells with high expression. In addition, we revealed the difference between the total mRNA degradation rate *γ*_*t*_ and spliced mRNA degradation rate *γ*_*s*_ based on their different physical roles, distinguished them in different models, and finally gave the relationship between these two (see “Methods” section). We noted that in Dynamo, to infer *β*, *γ*_*t*_ was first inferred when the splicing was ignored, then $\tilde{\gamma }≔\gamma_{s}/\beta$ was inferred using the method based on the steady-state assumption in scVelo [2], and finally $\gamma_{t}/\tilde{\gamma }$ was taken as the inference of *β* upon assuming *γ*_*t*_ = *γ*_*s*_. However, $\gamma_{t}/\tilde{\gamma }=\beta \gamma_{t}/\gamma_{s}$, while *γ*_*t*_ and *γ*_*s*_ are generally not equal. This point was overlooked in Dynamo, which causes an inaccurate estimate of *β*. In fact, under the steady-state assumption, *β* can be directly estimated by using only *u*_*l*_(*t*) through the formula *u*_*l*_(*t*) = (1 − *e*^−*βt*^)*α*/*β*, similar to the two-step method used in Dynamo to estimate *γ*_*t*_ through $l(t)=(1-e^{-\gamma_{t}t})\alpha /\gamma t$ since they have similar form. However, we don’t use this method in Storm.

**Fig 5:**
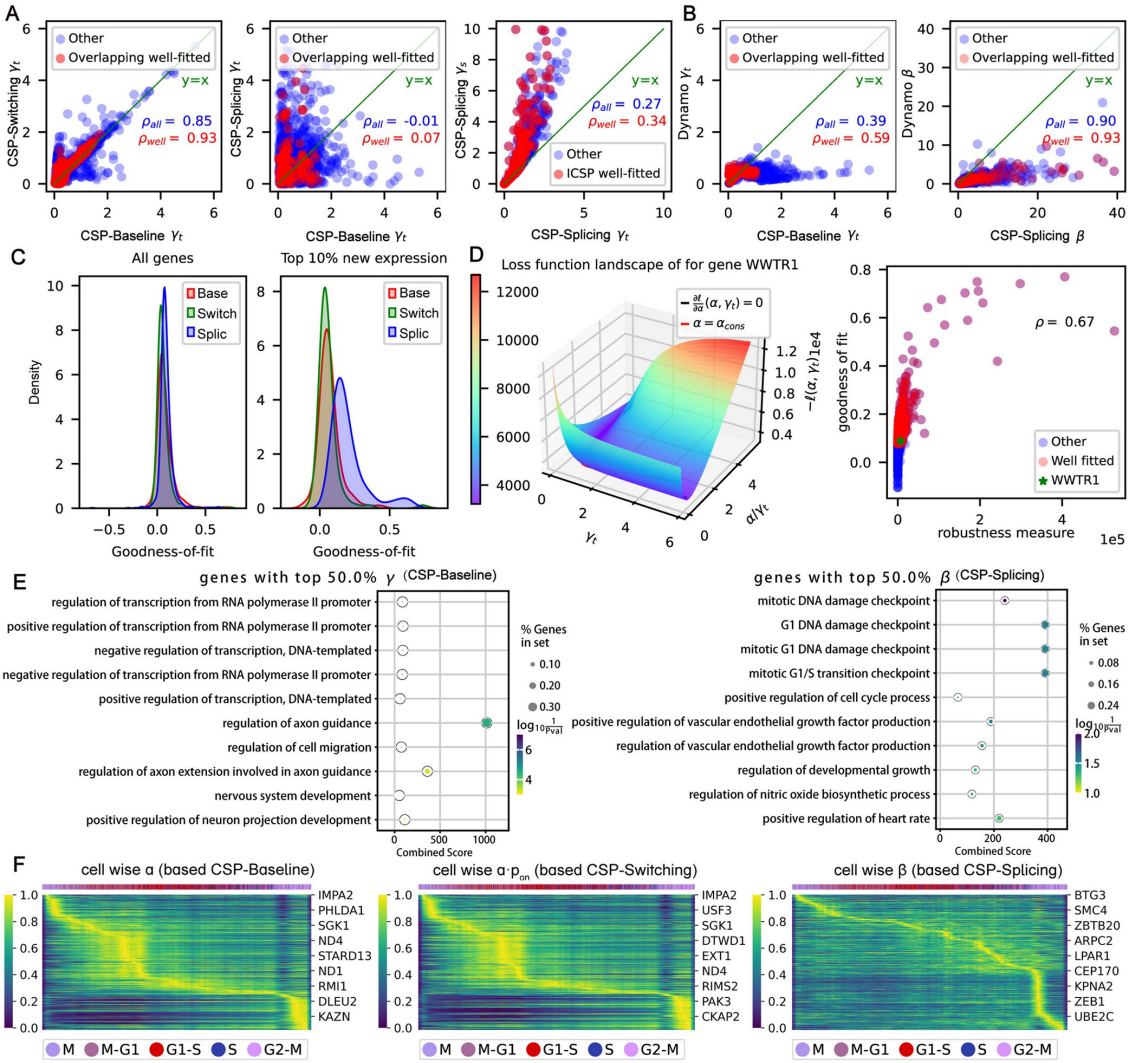
Parameter inference and enrichment analysis for the cell cycle dataset. **The inference strategy involved in this figure is for kinetics/pulse data**. **A.** Comparison of parameter inference results of our three stochastic models. From left to right are the comparison of *γ*_*t*_ of CSP-Baseline and CSP-Switching, the comparison of *γ*_*t*_ of CSP-Baseline and CSP-Splicing, the comparison of *γ*_*t*_ and *γ*_*s*_ in CSP-Splicing. The overlapping well-fitted genes were set as the overlap set of genes in the top 40% of the goodness-of-fit for both methods. **B.** Comparison of inferred parameters between our stochastic models and Dynamo’s method. **Left**: the comparison of *γ*_*t*_ between CSP-Baseline and Dynamo. **Right**: the comparison of *β* between CSP-Splicing and Dynamo. **C.** Comparison of the goodness-of-fit of the three stochastic models. **Left**: all highly variable genes. **Right**: genes in the top 10% of average new mRNA expression in highly variable genes. Here Base refers to the CSP-Baseline model, Splic to the CSP-Splicing model and Switch to the CSP-Switching model. **D.** Robust analysis. **Left**: Landscape of CSP-Baseline-based loss functions for the a typical gene *WWTR1*. **Right**: Scatter plot of robustness measure and goodness of fit for parameter inference. **E.** Enrichment analysis results of genes with high gene-wise *γ*_*t*_, *β* (top 50%) in well fitted genes (top 40% of goodness of fit). **F.** Heat map of cell-wise parameters for well-fitted genes. From left to right, cell-wise *α* based on the CSP-Baseline, cell-wise *αp*_on_ based on the CSP-Switching and cell-wise *β* based on the CSP-Splicing, respectively. Across all three heatmaps, the X-axis is the relative cell cycle position while the order of genes in the y-axis is arranged such that the peak time of each gene increases from the top left to bottom right.

With the above inference methods and insights, we studied a cell cycle dataset from the scEU-seq study [21]. We compared the parameter inference results of the three models (Fig 5A). When splicing was not considered, the inference results based on CSP-Baseline and CSP-Switching were close, with high correlation coefficients, especially in genes with higher goodness of fit (Fig 5A Left). However, whether or not splicing is considered significantly impacts the inference results. The inference results based on CSP-Baseline and CSP-Splicing were quite different, with low correlation coefficients, even in genes with higher goodness of fit (Fig 5A Middle). We speculate that this is due to the assumptions of the two models are incompatible: in CSP-Baseline, *γ*_*t*_ is assumed to be a constant; while in CSP-Splicing, *γ*_*s*_ is assumed to be a constant. But these two assumptions can not be held simultaneously for their different roles in the physical modeling and our analysis results (see “Methods” section). We also compared *γ*_*t*_ and *γ*_*s*_ computed by the CSP-Splicing, and the results showed that *γ*_*s*_ was always greater than *γ*_*t*_, and the linear correlation between the two was not high (Fig 5A Right). In summary, we showed that kinetic parameters inferred from CSP-Baseline and CSP-Switching but not CSP-Baseline and CSP-Splicing, are consistent.

The inferred total mRNA degradation rates *γ*_*t*_ from Storm and Dynamo are close in well-fitted genes, while CSP-Splicing’s inferred splicing rates *β* are always larger than those from Dynamo. We compared the inferred results of *γ*_*t*_ in CSP-Baseline with those in Dynamo (Fig 5B Left). Although they were not consistent for some genes, they are quite consistent for the genes with better fitting. We also compared the inference of *β* in CSP-Splicing with those in Dynamo (Fig 5B Right). The result shows that the inferred *β* by our approach was usually larger than those in Dynamo, even for the genes with a better fitting. A possible explanation is that the inference of Dynamo ignored the difference between *γ*_*t*_ and *γ*_*s*_, which made the inferred *β* smaller. We also compared the goodness-of-fit of the three stochastic models. Overall, they are relatively close (Fig 5C Left). However, when we focused on genes with higher new mRNA levels (top 10%), CSP-Splicing had a better fit (Fig 5C Right). We speculate that this is because genes with higher expression are suitable to be fitted with more complex models.

When the parameter *γ*_*t*_ is small, parameter inference may not be robust enough. However, we found that the genes selected by the goodness-of-fit have robust results. We analyzed the robustness of the parameter inference in the simplest CSP-Baseline model (see “Methods” section). We plotted the landscape of a typical negative log-likelihood loss function based on CSP-Baseline for gene *WWTR1* (Fig 5D Left), with the black line corresponding to ∂*ℓ*(*α*, *γ*_*t*_)/∂*α* = 0 (Eq (83) in the “Methods” section) and blue line corresponding to *α* = *α*_cons_ when $1-e^{-\gamma_{t}t}\sim \gamma_{t}t$ holds (Eq (84) in the “Methods” section). The landscape of the loss function shows a fairly flat area around ∂*ℓ*/∂*α* = 0, and the two lines almost coincide when *γ*_*t*_ is small, which is consistent with our previous argument. We design a quantitative index to measure the robustness of parameter inference (see “Methods” section) and analyzed the relationship between the robustness measure and the goodness-of-fit ${\bar{R}}_{D}^{2}$ (Fig 5D Right). We found that parameter robustness was positively correlated with the goodness of fit and the correlation coefficient was as high as 0.69. Though the reason for this high correlation is not clearly understood in theory, we can utilize this fact to select the genes with high goodness of fit for downstream analysis, which also ensures the results are relatively robust.

We selected the well-fitted genes (top 40% ${\bar{R}}_{D}^{2}$) and performed enrichment analysis on this fraction according to the magnitude of gene-wise parameters *γ*_*t*_, *β*, *α* and *p*_off_ (Fig 5E and S3 Fig). The results of the enrichment analysis showed that these genes were highly correlated with the cell cycle progression.

The assumption of constant coefficients is often violated because of the time-dependent kinetics and multiple lineages [11]. Many works relaxed the constant coefficient assumption and inferred cell-specific parameters to overcome this issue [10, 13, 16, 26]. In our proposal, we take a post-processing step to get the cell-specific parameters after inferring all parameters through previous procedures. We relaxed the constant coefficient assumption and proposed a method to infer cell-specific parameters except the constant degradation rate *γ*_*t*_ or *γ*_*s*_, i.e., we inferred cell-specific *α* in CSP-Baseline, cell-specific *α* × *p*_on_ in CSP-Switching, and the cell-specific *α* and *β* in CSP-Splicing (see “Methods” section). This partial constant coefficient assumption had support from the study in [21], which showed that the degradation rate of most genes was independent of time. Finally, We plotted heat maps of the cell-wise *α* (based on CSP-Baseline), *α* × *p*_on_ (based on CSP-Switching) and *β* (based on CSP-Splicing) for the well-fitted genes (Fig 5F). The results show that cells in the same cell cycle phase usually have closer kinetic parameters.

### Storm improves the robustness and accuracy of time-resolved RNA velocity analysis

Our three stochastic models described the evolution of the PMF (or joint PMF) of the number of new mRNA (or new unspliced and spliced mRNA) molecules over time for different settings. To estimate RNA velocity of single cells, only the evolution of the mean value over time will be considered, which requires us to reduce the stochastic models to the corresponding deterministic models (see “Methods” section).

Based on the deterministic model derived for the mean corresponding to the three stochastic models, we inferred the relevant parameters for computing different types of RNA velocity for different models. In Models 1 and 3, we computed the total RNA velocity $d\langle \tilde{r}\left(t\right)\rangle /dt$ because the splicing process was ignored. In CSP-Splicing, we calculated both total RNA velocity $d\langle \tilde{r}\left(t\right)\rangle /dt$ and spliced RNA velocity $d\langle \tilde{s}\left(t\right)\rangle /dt$ (see “Methods” section). Note that because the new RNA velocity mostly reflects the metabolic labeling process of RNA and does not reveal RNA biogenesis, it is thus not used. In addition, a derived relationship between *γ*_*t*_ and *γ*_*s*_ suggests that the total RNA velocity can be computed based on either $d\langle \tilde{r}\left(t\right)\rangle /dt=\alpha -\gamma_{s}\langle \tilde{s}\left(t\right)\rangle$ or $d\langle \tilde{r}\left(t\right)\rangle /dt=\alpha -\gamma_{t}\langle \tilde{r}\left(t\right)\rangle$. In practice, we used the former approach by default.

**Fig 6:**
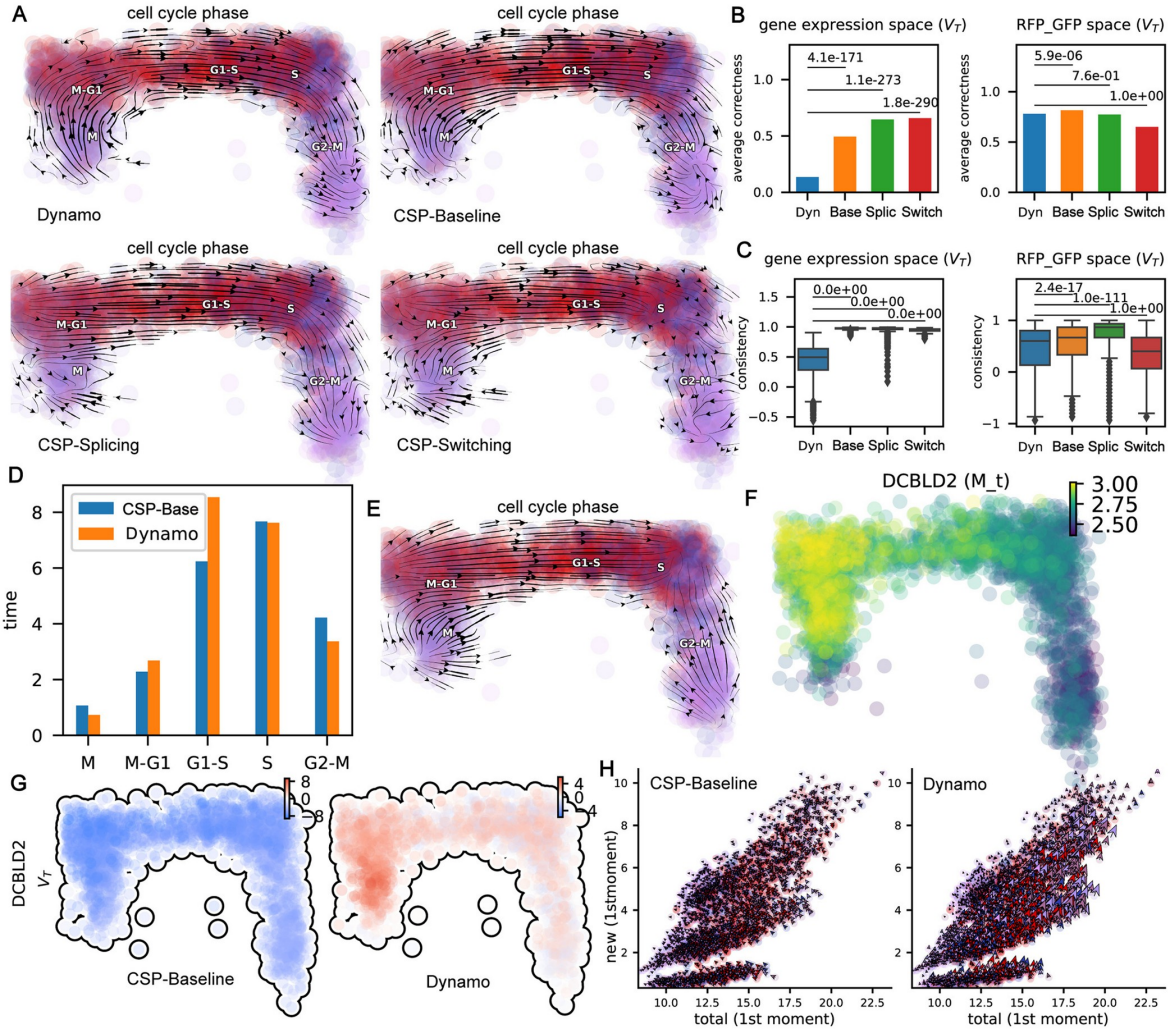
RNA velocity analysis of the cell cycle dataset. **The inference strategy involved in this figure is for kinetics/pulse data**. **A.** Comparison of total RNA velocity streamline visualizations between three stochastic methods and Dynamo in cell cycle dataset. **B.** Comparison of average correctness of total velocity in gene expression space and RFP_GFP space. The p-values are given by the one-sided Wilcoxon test. Here Base refers to the CSP-Baseline model, Splic to the CSP-Splicing model and Switch to the CSP-Switching model. **C.** Similar to **B**, comparison of velocity consistency. **D.** The duration time (unit: hour) of each cell cycle phase of the human RPE1-FUCCI system based on Storm’s CSP-Baseline and Dynamo. **E.** Total RNA velocity streamlines calculated using Storm’s CSP-Baseline with gene-wise parameters (instead of using gene-cell-wise parameters except for the degradation rate). **F.** The smoothed expression of *DCBLD2* in different cells. **G.** Comparison of total RNA velocity in *DCBLD2* between CSP-Baseline and Dynamo. **H.** Phase portraits of new-total RNA planes of *DCBLD2* of CSP-Baseline and Dynamo. Quivers correspond to the total (x-component) or new (y-component) RNA velocity calculated by the different methods.

We compared the streamlines of the total RNA velocity of our three models with that of Dynamo on the cell cycle scEU-seq dataset (Fig 6A). Almost all streamlines from our models correctly reflect the cell cycle progression, except that part of them from CSP-Splicing had a minor flaw in the M phase and CSP-Switching in the S phase. In addition, we found both CSP-Splicing and Dynamo’s spliced RNA velocity (S4A Fig) did not get entirely correct streamline results. The streamlines of CSP-Splicing were problematic in the M-G1 phase, while the streamlines of Dynamo were problematic in the S phase. We speculate that this is probably due to the fact that new unspliced mRNAs have rather low expression levels, frustrated with many dropouts and very sparse data, resulting in unreliable inferences of the parameter *β* and inaccurate RNA velocities.

We also quantitatively benchmarked the average correctness and consistency of the velocities in different methods in the original gene expression space and low-dimensional space (here the RFP_GFP space is used which corresponds to the Geminin-GFP and Cdt1-RFP-corrected signals of RPE1-FUCCI cells)(Fig 6B and 6C and S4B and S4C Fig). The definition of correctness and consistency of velocity is given in the “Methods” section. In the gene expression space, the average correctness and consistency of the total RNA velocity of CSP-Baseline, CSP-Splicing, and CSP-Switching are significantly better than that of Dynamo (Fig 6B and 6C Left), while the spliced RNA velocity of CSP-Splicing has slightly lower consistency than that of Dynamo (S4B and S4C Fig Left). In the RFP_GFP space, the average correctness of total RNA velocity of all methods are significantly higher compared to that in the gene expression space, and simpler methods tend to improve more. The average correctness of CSP-Baseline is highest at this time (Fig 6B Right). However, the average correctness of the CSP-Splicing’s spliced RNA velocity still perform slightly worse than Dynamo’s (S4B Fig Right). In contrast, the total RNA velocity consistency of CSP-Baseline and CSP-Splicing is significantly better than that of Dynamo (Fig 6C) and the spliced RNA velocity consistency of CSP-Splicing is also significantly better than that of Dynamo (S4C Fig Right). Overall, the CSP-Baseline-based total RNA velocity has the highest average correctness and consistency, and significantly outperforms Dynamo, while the CSP-Splicing-based spliced RNA velocity was close to Dynamo quantitatively.

To demonstrate the significance of inferring time-resolved velocities with physical units, we calculated the duration time of each cell cycle phase of the human RPE1-FUCCI system based on the total RNA velocities (see “Methods” section, Fig 6D). Indeed, the human RPE1-FUCCI system has a cell-cycle time of about 21 hours (about 6 hours for G1-S phase, 8 hours for S phase, 4 hours for G2-M phase, 1 hour for M phase and 2 hours for M-G1 phase) [37].

To demonstrate the value of using gene-cell-wise parameters (except degradation rates), we visualized the streamlines of total RNA velocity based on gene-cell-wise parameters and those based only on gene-wise parameters (Fig 6E and S4D Fig). We observed that the streamlines of CSP-Baseline and CSP-Switching in the S to G2-M phase are incorrectly reversed (Fig 6E and S4D Fig Right), and the streamlines of CSP-Splicing are also less smooth and accurate than those when gene-cell-wise parameters are used (S4D Fig Left).

We now illustrate the advantages of our method in the estimation of kinetic parameters and the calculation of RNA velocity with two example genes: *DCBLD2* and *HIPK2*. In gene *DCBLD2*, the cells at M and M-G1 have the highest overall expression and the correct RNA velocity should be negative (Fig 6F). However, Dynamo returned the positive velocity, which is problematic (Fig 6G Right). In contrast, CSP-Baseline, CSP-Switching and CSP-Splicing all returned negative velocities (Fig 6G Left and S4E Fig). We speculated one possible explanation is that the expression of the gene *DCBLD2* has not yet reached a steady state. Consistent results were also observed from phase portraits of new-total RNA planes of *DCBLD2* (Fig 6H and S4F Fig). For gene *HIPK2*, similarly, cells in phase M and M-G1 have the highest expression overall and the correct velocity should be negative (S4I Fig), but Dynamo and CSP-Baseline both returned positive velocities while CSP-Switching got the correct results (S4G and S4H Fig). We speculated one possible explanation for this is that the expression switch plays an important role in *HIPK2*.

Finally we generated a simulated non-steady-state pulse data (S5 Fig). Both the CSP-Baseline and Dynamo produced the correct streamlines (S5A Fig). However, the error between the degradation rate estimated by CSP-Baseline and the true value was lower compared to Dynamo, and the error was further reduced for the well-fitted genes selected by goodness-of-fit (S5B and S5C Fig).

## Discussion

Storm utilizes three stochastic models for the dynamical description of new mRNAs and allows the estimation of the RNA velocity for kinetics experiments and one-shot data with splicing information without the need for the steady-state assumption. It can also generally handle one-shot data without splicing information when the steady-state assumption is enforced. One possible limitation of our model is that it does not fully utilize the total mRNA information in kinetics experiments. According to the results of the chi-square independence test, the number of total mRNA molecules of most genes obeys the same distribution. Noting that the old mRNA molecules with a labeling duration of zero are the total mRNA molecules, we think that it is a feasible direction to establish the stochastic dynamics of old mRNA and use the Wasserstein distance in optimal transport approach [38, 39] to measure the differences between discrete distributions. Therefore, the optimal transport modeling of old RNAs may be integrated with Storm to obtain more robust RNA velocity inference. In addition, it is also worth exploring stochastic models that consider switching of gene expression states, transcription in the active state, splicing and spliced mRNA degradation simultaneously (i.e., integration of CSP-Splicing and CSP-Switching).

Some recent works, such as MultiVelo [7], Chromatin Velocity [40], and protaccel [8], extend RNA velocity to multi-omics. It is expected that the combination of metabolic labeling technology with other multi-omics measurements will bring new opportunities, which allows for simpler parameter inference and more accurate results.

Although Storm was able to infer cell-specific transcription and splicing rates through the post-processing steps, it still assumes that degradation rates are consistent across cells, which may introduce a potential bias. In addition, Storm like many methods assumes that genes are independent when inferring kinetic parameters (e.g. velocyto [1], scVelo [2], cellDancer [16], and Dynamo [26]), which is biologically implausible. Deep neural networks are expected to solve these problems by directly inferring cell-specific kinetic parameters and vector fields end-to-end in situations where gene regulation is considered. For example, DeepVelo [10] claims to achieve this goal for unspliced/spliced data. How to introduce deep neural networks to scRNA-seq data with metabolic labeling information is a direction worth exploring, and Storm may be able to provide some insights (e.g., loss function design) to achieve this goal.

Finally, Storm, like many other existing methods, first infers the RNA velocity in the high-dimensional gene expression space, then selects an appropriate two-dimensional embedding, and finally visualizes the RNA velocity by projecting it into the low-dimensional space. The two-step process was criticized and may lead to specious results [6, 41–43]. Nevertheless, a large number of efforts have been proposed to compensate for the shortcomings of the two-step process of projecting gene-specific RNA velocities from high-dimensional space to low-dimensional embeddings. For example, UnitVelo [9] supports the inference of a unified latent time across the transcriptome, GraphDynamo [44] maps the cellular dynamics onto a discrete graph representation, PAGA [45] generates a much simpler abstracted directed graph of partitions by using RNA velocity in raw space, and LatentVelo [15], DeepCycle [46] and VeloCycle [47] simultaneously infer the hidden space of gene expression and the dynamics on the hidden space, where DeepCycle [46] and VeloCycle [47] are also specifically designed for cell cycle processes. We tested Storm’s inference results using PAGA. The results show that Storm’s stochastic modeling strategy is effective (S6 Fig). However, we would also like to mention that the determination of which visualization is the best choice is not an easy problem. A thorough discussion about this issue deserves independent publications and detailed comparisons and studies in the future, which is not the main concern of Storm. The key contribution of Storm is the design of parameter inference methods for the scRNA-seq data with metabolic labeling that does not rely on steady-state assumptions.

## Conclusion

We present Storm for estimating absolute kinetic parameters and inferring the time-resolved RNA velocity of metabolic labeling scRNA-seq data by incorporating the transient stochastic dynamics of gene expressions. Storm establishes three stochastic models of new mRNA which take into account both biological noise and cell-specific technical noise, and makes inference to the gene-specific degradation rates and other gene-cell-specific parameters without relying on the steady-state assumption in kinetics experiments and one-shot data with splicing information. It can also handle one-shot data without splicing information when the steady-state assumption is adopted. Numerical results show that Storm is able to accurately fit the kinetic cell cycle dataset and many one-shot experimental datasets. In addition, our numerical experience suggests that CSP-Baseline outperforms the other two models when splicing dynamics is not of interest, and CSP-Splicing is the valid choice if the data contains both labeling and splicing information and splicing dynamics is of interest. However, further applications and performance evaluations for more challenging datasets with temporal information are desired and it will be studied in the future. We hope the developed method will become increasingly important when more metabolic labeling data are available.

## Methods

### Derivation of three stochastic dynamical models

Here we developed three stochastic models for the dynamical description of new mRNAs: Model 1 (CSP-Baseline): a stochastic dynamical model of new mRNA involving only metabolic-labeling transcription and degradation; Model 2 (CSP-Splicing): a stochastic dynamical model of new unspliced and spliced mRNA involving metabolic-labeling transcription, splicing and spliced mRNA degradation; and Model 3 (CSP-Switching): a stochastic dynamical model of new mRNA involving gene state switching, metabolic-labeling transcription and degradation.

#### Model 1 (CSP-Baseline): Stochastic dynamical modeling of new mRNA

Following [21, 26], we made the following assumptions: (1) Genes are independent. (2) Both the transcription rate *α* and the degradation rate of total mRNA *γ*_*t*_ are constants.

The chemical master equation (CME) for the new/labeled mRNA $\tilde{l}\left(t\right)$, corresponding to the chemical reactions shown in the first row of Fig 1A, is given by

$$\begin{array}{c} \frac{d{\tilde{P}}_{n}}{dt}=-\left(\alpha +n\gamma_{t}\right){\tilde{P}}_{n}+\alpha {\tilde{P}}_{n-1}+\gamma_{t}\left(n+1\right){\tilde{P}}_{n+1}, \end{array}$$ (5)

where ${\tilde{P}}_{n}\left(t\right)=\text{Prob}\left(\tilde{l}\left(t\right)=n\right)$. The initial value of new mRNA count is zero, i.e., ${\tilde{P}}_{n}\left(0\right)=\delta_{0n}$, where

$$\delta_{mn}=\{\begin{array}{cc} 1, & \;\text{if}\;m=n\\ 0, & \;\text{otherwise}\; \end{array}$$

is the Kronecker’s delta function. The solution of Eq (5) is

$$\begin{array}{c} {\tilde{P}}_{n}\left(t\right)=\frac{a{\left(t\right)}^{n}}{n!}e^{-a(t)},\;n\in N, \end{array}$$ (6)

where $a(t)=\alpha (1-e^{-\gamma_{t}t})/\gamma t$. This means that $\tilde{l}\left(t\right)$ obeys the Poisson distribution with mean *a*(*t*).

The above stochastic model only describes the true expression count of new mRNA $\tilde{l}\left(t\right)$ in a cell with labeling duration *t*, but the measured sequencing data is different from this count due to technical noise. Denote by *l*(*t*) the number of measured new mRNA molecules, and assume that *l*(*t*) is associated with $\tilde{l}\left(t\right)$ through a dropout process, which we modeled as a binomial distribution:

$$\begin{array}{c} \text{Prob}\left(l\left(t\right)=n|\tilde{l}\left(t\right)=N\right)=C_{N}^{n}p^{n}{\left(1-p\right)}^{N-n}≔B_{n}\left(N,p\right), \end{array}$$ (7)

where *p* is the capture probability of a single mRNA molecule. We further assume that the total number of mRNA molecules across all genes in different cells are close, which was commonly adopted in the preprocessing step [1, 2, 26]. Denote by *n*_*j*_ the total number of mRNA molecules across all genes in cell *j*, i.e., *n*_*j*_ = ∑_*i*_ *r*_*ij*_, where *r*_*ij*_ refers to the number of mRNA molecules in gene *i* of cell *j* in the scRNA-seq measurements. This assumption implies that the capture probability of mRNA molecules in different cells is different, and *p*_*j*_ ∝ *n*_*j*_. In our computation, we took *p*_*j*_ = *n*_*j*_/*n*_0_, where *n*_0_ = *n*_med_ is the median of *n*_*j*_. Note that *p*_*j*_ here is what is commonly referred to as the size factor, which is chosen to be consistent with the deterministic approach. Such choice might make *p*_*j*_ > 1 for some *j*. However, this artifact can be easily avoided by taking *n*_0_ larger, e.g., ${\tilde{n}}_{0}≔{\text{max}}_{j}\;n_{j}$. This alternative choice does not affect the inference of the degradation and splicing rates except that the transcription rate *α* will be rescaled by the multiple ${\tilde{n}}_{0}/n_{\text{med}}$ from Eq (9) and the form of *a*(*t*). In this case, the direction of the inferred RNA velocity is not affected up to a common multiplicative constant, and the whole approach is still valid.

We denoted the PMF of new mRNA sequencing result *l*_*j*_(*t*) of cell *j* with labeling duration *t* by

$$\begin{array}{c} P_{n,j}\left(t\right)≔\text{Prob}(l_{j}\left(t\right)=n). \end{array}$$ (8)

Then

$$\begin{array}{c} P_{n,j}\left(t\right)=\sum_{N=n}^{\infty }{\tilde{P}}_{N}\left(t\right)B_{n}(N,p_{j})=\frac{{\left(p_{j}a\left(t\right)\right)}^{n}}{n!}e^{-p_{j}a\left(t\right)}, \end{array}$$ (9)

which means that *l*_*j*_(*t*) obeys the Poisson distribution with mean *p*_*j*_*a*(*t*).

In summary, the former derivation shows that the number of new mRNA molecules in different cells in scRNA-seq measurements obeys Poisson distribution with cell-specific parameters, and these parameters were proportional to *p*_*j*_, i.e., proportional to *n*_*j*_. We call this distribution the *cell-specific Poisson distribution*.

#### Model 2 (CSP-Splicing): Stochastic dynamical modeling of new unspliced and spliced mRNAs

Compared with CSP-Baseline, we distinguished whether an mRNA molecule is spliced or not and incorporated the splicing process, which was shown in the second row of Fig 1A. Again we assumed that the genes are independent. In addition, we further assumed that the transcription rate *α*, splicing rate *β*, and spliced mRNA degradation rate *γ*_*s*_ are all constants.

The CME for the new/labeled unspliced and spliced mRNAs $\left({\tilde{u}}_{l}\left(t\right),{\tilde{s}}_{l}\left(t\right)\right)$, corresponding to the considered chemical reactions shown in the second row of Fig 1A, is given by

$$\begin{array}{cc} \partial_{t}{\tilde{P}}_{mn} & =\alpha \left({\tilde{P}}_{m-1,n}-{\tilde{P}}_{mn}\right)+\beta [\left(m+1\right){\tilde{P}}_{m+1,n-1}-m{\tilde{P}}_{mn}]\\ & +\gamma_{s}[\left(n+1\right){\tilde{P}}_{m,n+1}-n{\tilde{P}}_{mn}], \end{array}$$ (10)

where ${\tilde{P}}_{mn}\left(t\right)=\text{Prob}\left(\left({\tilde{u}}_{l}\left(t\right),{\tilde{s}}_{l}\left(t\right)\right)=\left(m,n\right)\right)$. The initial distribution of new unspliced and spliced mRNA is ${\tilde{P}}_{mn}\left(0\right)=\delta_{m0}\delta_{n0}$. The solution of Eq (10) is

$$\begin{array}{c} {\tilde{P}}_{mn}\left(t\right)=b{\left(t\right)}^{m}c{\left(t\right)}^{n}e^{-b(t)-c(t)}/m!n!,\;\left(m,n\right)\in N^{2}, \end{array}$$ (11)

where

$$\begin{array}{c} \begin{array}{cc} b(t) & =\alpha (1-e^{-\beta t})/\beta ,\\ c(t) & =\{\begin{array}{cc} \frac{\alpha }{\gamma_{s}}\left(1-e^{-\gamma_{s}t}\right)+\frac{\alpha }{\gamma_{s}-\beta }\left(e^{-\gamma_{s}t}-e^{-\beta t}\right), & \beta \neq \gamma_{s},\\ \frac{\alpha }{\beta }\left(1-e^{-\beta t}\right)-\alpha te^{-\beta t}, & \beta =\gamma_{s}, \end{array} \end{array} \end{array}$$ (12)

which means that ${\tilde{u}}_{l}\left(t\right)$ and ${\tilde{s}}_{l}\left(t\right)$ obey independent Poisson distributions with mean *b*(*t*) and *c*(*t*), respectively. We refer interested readers to [3] for derivation details.

Denote by (*u*_*l*_(*t*), *s*_*l*_(*t*)) the number of measured new unspliced and spliced mRNA molecules in the scRNA-seq experiments with labeling duration *t*. By assuming that the dropout processes for new unspliced and spliced mRNAs are independent and the capture probability is independent of whether they are spliced or not, we modeled the dropout process for ${\tilde{u}}_{l}\left(t\right)$ and ${\tilde{s}}_{l}\left(t\right)$ as independent binomial distributions with the same parameter *p*. So we got

$$\begin{array}{cc} \text{Prob} & \left(\left(u_{l}\left(t\right),s_{l}\left(t\right)\right)=\left(m,n\right)|\left({\tilde{u}}_{l}\left(t\right),{\tilde{s}}_{l}\left(t\right)\right)=\left(M,N\right)\right)\\ & =C_{M}^{m}p^{m}{\left(1-p\right)}^{M-m}C_{N}^{n}p^{n}{\left(1-p\right)}^{N-n}≔B_{m}\left(M,p\right)B_{n}\left(N,p\right). \end{array}$$ (13)

For the same reason as CSP-Baseline, we take *p*_*j*_ proportional to *n*_*j*_. And we took *p*_*j*_ = *n*_*j*_/*n*_med_ in the computation.

We denoted the joint PMF of new unspliced and spliced mRNA sequencing counts (*u*_*l*,*j*_(*t*), *s*_*l*,*j*_(*t*)) of cell *j* with labeling duration *t* by

$$P_{mn,j}\left(t\right)≔\text{Prob}((u_{l,j}\left(t\right),s_{l,j}\left(t\right))=\left(m,n\right)).$$

Then

$$\begin{array}{cc} P_{mn,j}\left(t\right) & =\sum_{M=m}^{\infty }\sum_{N=n}^{\infty }\frac{b{\left(t\right)}^{M}c{\left(t\right)}^{N}}{M!N!}e^{-b(t)-c(t)}B_{M}\left(m,p_{j}\right)B_{N}\left(n,p_{j}\right)\\ & =\sum_{M=m}^{\infty }\frac{b{\left(t\right)}^{M}}{M!}e^{-b(t)}B_{M}\left(m,p_{j}\right)\sum_{N=n}^{\infty }\frac{c{\left(t\right)}^{N}}{N!}e^{-c(t)}B_{N}\left(n,p_{j}\right)\\ & =\frac{{\left(p_{j}b\left(t\right)\right)}^{m}}{m!}e^{-p_{j}b\left(t\right)}\frac{{\left(p_{j}c\left(t\right)\right)}^{n}}{n!}e^{-p_{j}c\left(t\right)}, \end{array}$$ (14)

which means that *u*_*l*,*j*_(*t*) and *s*_*l*,*j*_(*t*) are independently Poisson distributed with mean *p*_*j*_*b*(*t*) and *p*_*j*_*c*(*t*), respectively.

In summary, (*u*_*l*_(*t*), *s*_*l*_(*t*)) obeys independent cell-specific Poisson distribution.

#### Model 3 (CSP-Switching): Stochastic dynamical modeling of new mRNA considering switching

In CSP-Switching, we further considered the on/off gene state switching shown in the third row of Fig 1A. We assumed that the genes are independent as well, and the transcription rate *α*, mRNA degradation rate *γ*_*t*_, the gene on-to-off rate *k*_off_ and off-to-on rate *k*_on_ are all constants. Furthermore, following [32] we assumed that *k*_on_ and *k*_off_ are significantly smaller than *α* and *γ*_*t*_, which implies that the gene expression is either always on or always off during the transcription/degradation period. From Eq (5), it is known that cells in the on state obey a Poisson distribution with mean *a*(*t*), while cells in the off state do not express. Define *p*_off_ = *k*_off_/(*k*_on_ + *k*_off_). Then $\tilde{l}\left(t\right)$ obeys the zero-inflated Poisson distribution

$$\begin{array}{c} \begin{array}{cc} {\tilde{P}}_{0}\left(t\right) & =\left(1-p_{\text{off}}\right)e^{-a(t)}+p_{\text{off}},\\ {\tilde{P}}_{n}\left(t\right) & =\left(1-p_{\text{off}}\right)\frac{a{\left(t\right)}^{n}}{n!}e^{-a(t)},\;\;n\geq 1. \end{array} \end{array}$$ (15)

Similarly, by taking into account the technical noise in scRNA-seq experiments, the PMF of *l*_*j*_(*t*) is

$$\begin{array}{c} \begin{array}{cc} P_{0,j}\left(t\right) & =\left(1-p_{\text{off}}\right)e^{-p_{j}a\left(t\right)}+p_{\text{off}},\\ P_{n,j}\left(t\right) & =\left(1-p_{\text{off}}\right)\frac{{\left(p_{j}a\left(t\right)\right)}^{n}}{n!}e^{-p_{j}a\left(t\right)},\;\;n\geq 1. \end{array} \end{array}$$ (16)

In summary, different cells obey the ZIP distribution with different parameters as shown in Eq (16), which we called cell-specific zero-inflated Poisson distribution.

### Chi-square goodness-of-fit test for cell-specific distributions at a fixed time

We would construct an asymptotic *χ*^2^ statistic for the data with common distribution type but sample-specific parameters. This goodness-of-fit test is to assess whether the null hypothesis that the considered data, at a fixed labeling duration, obeys the proposed distribution can be accepted.

We first divided the value range of the considered data into *c* classes. According to the range that the samples fall in, we got *n* independent categorically distributed random samples *X*_*i*_ ∈ {1, 2, …, *c*} for *i* = 1, 2, …, *n* with sample dependent parameter *p*_*i*_, respectively. An equivalent representation for the categorical variable *X*_*i*_ is to denote *X*_*i*_ = (*X*_*ij*_)_*j* = 1, …, *c*_ ∈ {*e*_1_, …, *e*_*c*_}, where *e*_*j*_ = (*δ*_*jk*_)_*k* = 1, …, *c*_ is the indicator vector for *j* = 1, …, *c*. Correspondingly, the parameter *p*_*i*_ = (*p*_*i*1_, …, *p*_*ic*_)^*T*^ is a *c*-dimensional vector with non-negative elements and sums to one, which is defined as

$$\begin{array}{c} p_{ij}≔\text{Prob}\left(X_{ij}=1\right)=1-\text{Prob}\left(X_{ij}=0\right),\;j=1,\ldots ,c. \end{array}$$ (17)

This implies that Var(*X*_*ij*_) = *p*_*ij*_(1 − *p*_*ij*_) and $\text{Cov}\left(X_{ij},X_{il}\right)=E\left[X_{ij}X_{il}\right]-p_{j}p_{l}=-p_{j}p_{l}\;\text{for}\;j\neq l$. Therefore, the covariance matrix of random vector *X*_*i*_ is

$$\begin{array}{c} \Sigma_{i}=(\begin{array}{cccc} p_{i1}\left(1-p_{i1}\right) & -p_{i1}p_{i2} & \ldots & -p_{i1}p_{ic}\\ -p_{i1}p_{i2} & p_{i2}\left(1-p_{i2}\right) & \ldots & -p_{i2}p_{ic}\\ \vdots & \vdots & \ddots & \vdots \\ -p_{i1}p_{ic} & -p_{i2}p_{ic} & \ldots & p_{ic}\left(1-p_{ic}\right) \end{array}). \end{array}$$ (18)

For sample *i*, we defined the truncated random vector $X_{i}^{*}={\left(X_{i1},\ldots ,X_{i,c-1}\right)}^{T}$ and truncated vector $p_{i}^{*}={\left(p_{i1},\ldots ,p_{i,c-1}\right)}^{T}$, which is the first *c* − 1 components of *X*_*i*_ and *p*_*i*_, respectively. The covariance matrix of $X_{i}^{*}$ is the submatrix consisting of the upper-left (*c* − 1) × (*c* − 1) block of Σ_*i*_, denoted by $\Sigma_{i}^{*}$, which can be written as

$$\begin{array}{c} \Sigma_{i}^{*}=\text{diag}\left(p_{i}^{*}\right)-p_{i}^{*}{\left(p_{i}^{*}\right)}^{T}, \end{array}$$ (19)

where $\text{diag}(p_{i}^{*})$ is the diagonal matrix formed by the components of $p_{i}^{*}$.

Define ${\bar{X}}^{*}≔\left(\sum_{i=1}^{n}X_{i}^{*}\right)/n$, ${\bar{p}}^{*}≔\left(\sum_{i=1}^{n}p_{i}^{*}\right)/n$ and ${\bar{\Sigma }}^{*}≔\left(\sum_{i=1}^{n}\Sigma_{i}^{*}\right)/n$, and let

$$\begin{array}{c} \chi^{2}≔n{\left({\bar{X}}^{*}-{\bar{p}}^{*}\right)}^{T}{\left({\bar{\Sigma }}^{*}\right)}^{-1}\left({\bar{X}}^{*}-{\bar{p}}^{*}\right). \end{array}$$ (20)

Below we would show that *χ*^2^ is an asymptotic chi-square statistic with degrees of freedom *c* − 1. First note that

$$\begin{array}{c} \text{E}\left[{\bar{X}}^{*}\right]=\text{E}[\frac{1}{n}\sum_{i=1}^{n}X_{i}^{*}]=\frac{1}{n}\sum_{i=1}^{n}\text{E}\left[X_{i}^{*}\right]=\frac{1}{n}\sum_{i=1}^{n}\;p_{i}^{*}={\bar{p}}^{*}, \end{array}$$ (21)

then the covariance

$$\begin{array}{c} \text{D}\left[{\bar{X}}^{*}\right]=\text{D}[\frac{1}{n}\sum_{i=1}^{n}X_{i}^{*}]=\frac{1}{n^{2}}\sum_{i=1}^{n}\text{D}\left[X_{i}^{*}\right]=\frac{1}{n}(\frac{1}{n}\sum_{i=1}^{n}\Sigma_{i}^{*})=\frac{1}{n}{\bar{\Sigma }}^{*}. \end{array}$$ (22)

Let $Y_{n}=\sqrt{n}{\left(\Sigma^{*}\right)}^{-1/2}\left({\bar{X}}^{*}-{\bar{p}}^{*}\right)$. When *n* goes to infinity, *Y*_*n*_ converges in distribution to the normal distribution *N*(0, *I*_*c*−1_) according to the central limit theorem for the independent sum of random variables. Thus, $\chi^{2}=Y_{n}^{T}Y_{n}$ converges in distribution to a chi-square distribution with degrees of freedom *c* − 1.

In summary, we proposed a new asymptotic *χ*^2^ statistic for sample-specific distributions. For a fixed labeling duration *t*_fixed_, *a*(*t*_fixed_), *b*(*t*_fixed_) and *c*(*t*_fixed_) are all constants, the proposed *χ*^2^ statistics can be used to test whether the new mRNA sequencing data are consistent with the CSP, ICSP and CSZIP distributions based on Models CSP-Baseline, CSP-Splicing and CSP-Switching, respectively. In addition, since there are one, two and two parameters to be inferred in CSP, ICSP and CSZIP distributions, respectively, the same number of degrees of freedom should be subtracted. Following [28], we ensured that the expected count *np*_*j*_ ≥ 0.25 in each group when determining the group value ranges. Finally, we take *p*-value as 0.05 in the computation.

### Parameter inference in one-shot experiments with steady state assumption

In the one-shot experiments where we only observe new RNA *l*_*j*_(*t*) and total RNA *r*_*j*_(*t*) data for one labeling duration *t*, we had to invoke the steady-state assumption for the total RNA.

When the dynamics of total RNA in CSP-Baseline is at steady state, i.e.,

$$\begin{array}{c} 0=\frac{d{\tilde{P}}_{r,n}}{dt}=-\left(\alpha +n\gamma_{t}\right){\tilde{P}}_{r,n}+\alpha {\tilde{P}}_{r,n-1}+\gamma_{t}\left(n+1\right){\tilde{P}}_{r,n+1}, \end{array}$$ (23)

where ${\tilde{P}}_{r,n}≔\text{Prob}(\tilde{r}=n)$ is the invariant PMF of the true expression of total RNA. At this point ${\tilde{P}}_{r,n}$ is a Poisson distribution with *α*/*γ*_*t*_ as the mean and from [3] we know that at this point the PMF of the true expression of old RNA ${\tilde{P}}_{u,n}$ is a Poisson distribution with $(\alpha /\gamma t)e^{-\gamma_{t}t}$ as the mean. From Eq (9) we know that when technical noise is considered, the observed old RNA counts obey a similar CSP distribution

$$\begin{array}{c} P_{u,n,j}=\frac{{\left(p_{j}\left(\alpha /\gamma_{t}\right)e^{-\gamma_{t}t}\right)}^{n}}{n!}e^{-p_{j}\left(\alpha /\gamma_{t}\right)e^{-\gamma_{t}t}}. \end{array}$$ (24)

At this point, we obtained the distributions of the new RNA and old RNA observations so that parameter inference can be performed using the MLE. Notice that the distribution of new RNA counts is not independent of total RNA counts, whereas the distribution of new RNA and old RNA counts is independent. We want to maximize the log-likelihood function

$$\begin{array}{cc} \ell (\alpha ,\gamma_{t}) & =\sum_{j=1}^{n}\;\text{log}\;(\text{Prob}(r=r_{j},l=l_{j}))\\ & =\sum_{j=1}^{n}\;\text{log}\;(\text{Prob}(u=r_{j}-l_{j},l=l_{j}))\\ & =\sum_{j=1}^{n}\;\text{log}\;(P\left(p_{j}a\left(t\right)\right){|}_{l_{j}})+\text{log}\;(P(p_{j}\left(\alpha /\gamma_{t}\right)e^{-\gamma_{t}}){|}_{r_{j}-l_{j}}) \end{array}$$ (25)

where ${P\left(\lambda \right)|}_{n}≔\text{Prob}\left(X=n\right)=e^{-\lambda }\lambda^{n}/n!$ is the probability of *X* = *n* for a Poisson-distributed random variable *X* with mean λ. When ∂*ℓ*/∂*α* = 0 and ∂*ℓ*/∂*γ*_*t*_ = 0, the likelihood function is maximized and it can be solved analytically

$$\begin{array}{c} \gamma_{t}=-\frac{1}{t}\;\text{log}\;(1-\frac{\left\langle l_{j}\right\rangle }{\left\langle r_{j}\right\rangle }),\;\alpha =\gamma_{t}\frac{\left\langle r_{j}\right\rangle }{\left\langle p_{j}\right\rangle }, \end{array}$$ (26)

where 〈⋅〉 means the population average defined by

$$\begin{array}{c} \left\langle \cdot \right\rangle =(\sum_{k=1}^{K}\sum_{j=1}^{n_{k}}\left(\cdot \right))/(\sum_{k=1}^{K}n_{k}). \end{array}$$ (27)

Since here it is for the one-shot data set, *K* = 1. Note that Eq (26) is similar to the formula in Dynamo [26] for estimating the parameters for one-shot data. The difference is that this formula averages the raw counts, while the method in Dynamo averages the smoothed data.

### Parameter inference in one-shot experiments without steady state assumption

In one-shot dataset containing both labeling and splicing information, i.e. unspliced unlabeled RNA *u*_*u*,*j*_, unspliced labeled RNA *u*_*l*,*j*_, spliced unlabeled RNA *s*_*u*,*j*_ and spliced labeled RNA *s*_*l*,*j*_ information is observed, we can make parametric inference without relying on the steady-state assumption.

The method is divided into two steps; in the first step, we sum unspliced unlabeled RNA and unspliced labeled RNA to obtain unspliced RNA, and unspliced labeled RNA and spliced labeled RNA to obtain spliced RNA. Then, we use the dynamic model without relying on steady state assumption in scVelo proposed by Bergen et al. [2] to infer the observation time of cells *t*_obs,*j*_, switching time *t*_*s*_, degradation rate *γ*_*s*,scv_, and splicing rate *β*_scv_. Despite the problem of scale invariance, the absolute magnitude of these values is not physically meaningful, but can still provide useful information for inferring the absolute magnitude of the parameters, e.g., the value of *β*_scv_/*γ*_*s*,scv_ is meaningful, and in addition the cells with *t*_obs,*j*_ less than *t*_*s*_ are in the on state.

In the second step, we integrate the results from the first step and the labeling information to determine the absolute size of the parameter. We define the notation

$$S_{\text{on}}≔\left\{j|t_{\text{obs},j}<t_{s}\right\}$$

to be the set of cells in the on state. In CSP-Splicing, the *ul*_*j*_ and *sl*_*j*_ of cells in the on state obey ICSP distribution

$$\begin{array}{c} P_{mn,j}\left(t\right)=\frac{{\left(p_{j}b\left(t\right)\right)}^{m}}{m!}e^{-p_{j}b\left(t\right)}\frac{{\left(p_{j}c\left(t\right)\right)}^{n}}{n!}e^{-p_{j}c\left(t\right)},\;\forall \;j\in S_{\text{on}}. \end{array}$$ (28)

We want to maximize the log-likelihood function

$$\begin{array}{c} \ell \left(\alpha ,\beta ,\gamma_{s}\right)=\sum_{j\in S_{\text{on}}}\;\text{log}\;(P\left(p_{j}\left(t\right)b\left(t\right)\right){{|}_{u_{l,j}\left(t\right)}\cdot P\left(p_{j}\left(t\right)c\left(t\right)\right)|}_{s_{l,j}\left(t\right)}), \end{array}$$ (29)

which is equivalent to

$$\begin{array}{cc} \frac{\sum_{j\in S_{\text{on}}}u_{l,j}}{\sum_{j\in S_{\text{on}}}p_{j}} & =b\left(t\right)=\frac{\alpha }{\beta }\left(1-e^{-\beta t}\right),\\ \frac{\sum_{j\in S_{\text{on}}}s_{l,j}}{\sum_{j\in S_{\text{on}}}p_{j}} & =c\left(t\right)=\frac{\alpha }{\gamma_{s}}\left(1-e^{-\gamma_{s}t}\right)+\frac{\alpha }{\gamma_{s}-\beta }\left(e^{-\gamma_{s}t}-e^{-\beta t}\right). \end{array}$$ (30)

This problem is not well-defined, but it is after adding the result

$$\begin{array}{c} \frac{\beta }{\gamma_{s}}=\frac{\beta_{\text{scv}}}{\gamma_{s,\text{scv}}} \end{array}$$ (31)

from the first step. By solving the system of nonlinear equations consisting of equations (30) and (31), we can obtain the absolute magnitudes of *α*, *β*, and *γ*_*s*_.

Since modeling assumptions in scVelo are often violated, we selected only well-fitting genes for use in the second stage and downstream analyses. We use *R*^2^ as the goodness-of-fit, which is defined as

$$\begin{array}{c} R^{2}=1-\frac{\sum_{j=1}^{N}{\left(\left(u_{j},s_{j}\right)-\left(u\left(t_{\text{obs},j}\right),s\left(t_{\text{obs},j}\right)\right)\right)}^{2}}{\sum_{j=1}^{N}{\left(\left(u_{j},s_{j}\right)-\left(\bar{u},\bar{s}\right)\right)}^{2}}. \end{array}$$ (32)

Due to the dropout effect, cells with expression close to 0 are not used in the actual calculation of *R*^2^. More specifically the rule is (*u*_*j*_, *s*_*j*_) < (*max*(*u*_*j*_)/5, *max*(*s*_*j*_)/5).

### Parameter inference in kinetics experiments

In the kinetics experiments, we observed data *l*_*j*_(*t*_*k*_) (or (*u*_*l*,*j*_(*t*_*k*_), *s*_*l*,*j*_(*t*_*k*_))) for new mRNA (or new unspliced and spliced mRNAs) with different labeling durations. We assumed that there are *K* labeling durations *t*_*k*_ for *k* = 1, 2, …, *K*, and the number of cells with labeling duration *t*_*k*_ is *n*_*k*_. We utilized the MLE to infer the unknown parameters in different models without relying on steady-state assumptions.

In CSP-Baseline, we need to maximize the log-likelihood function

$$\begin{array}{c} \ell \left(\alpha ,\gamma_{t}\right)=\sum_{k=1}^{K}\sum_{j=1}^{n_{k}}\;\text{log}\;(P\left(p_{j}\left(t_{k}\right)a\left(t_{k}\right)\right){|}_{l_{j}\left(t_{k}\right)}). \end{array}$$ (33)

It is equivalent to minimizing the following loss function

$$\begin{array}{c} L\left(\alpha ,\gamma_{t}\right)=\sum_{k=1}^{K}\sum_{j=1}^{n_{k}}-l_{j}\left(t_{k}\right)\;\text{log}\left(p_{j}\left(t_{k}\right)a\left(t_{k}\right)\right)+p_{j}\left(t_{k}\right)a\left(t_{k}\right). \end{array}$$ (34)

The optimum of the loss is achieved when the gradient equals 0. Utilizing the concrete expression of *a*(*t*) in CSP-Baseline, we got $\partial a(t)/\partial \alpha =(1-e^{-\gamma_{t}t})/\gamma t$. Then ∂*L*(*α*, *γ*_*t*_)/∂*α* = 0 has a closed form solution

$$\begin{array}{c} \alpha \left(\gamma_{t}\right)=\frac{\left\langle l_{j}\left(t_{k}\right)\right\rangle }{\left\langle p_{j}\left(t_{k}\right)\partial a\left(t_{k}\right)/\partial \alpha \right\rangle }. \end{array}$$ (35)

Another component of the Euler-Lagrange equation ∂*L*/∂*γ*_*t*_ = 0 has no closed form solution, so we need to solve *γ*_*t*_ by numerical iterations. We took the initial value of *γ*_*t*_ as the solution from Dynamo [26] under the steady-state assumption. Denote it as *γ*_*t*,0_, and correspondingly, we take the initial value of *α* as *α*_0_ = *α*(*γ*_*t*,0_).

In CSP-Splicing, we need to maximize the log-likelihood function

$$\begin{array}{c} \ell \left(\alpha ,\beta ,\gamma_{s}\right)=\sum_{k=1}^{K}\sum_{j=1}^{n_{k}}\;\text{log}\;(P\left(p_{j}\left(t_{k}\right)b\left(t_{k}\right)\right){{|}_{u_{l,j}\left(t_{k}\right)}\cdot P\left(p_{j}\left(t_{k}\right)c\left(t_{k}\right)\right)|}_{s_{l,j}\left(t_{k}\right)}), \end{array}$$ (36)

which is equivalent to minimizing the loss function

$$\begin{array}{cc} L(\alpha ,\beta ,\gamma_{s}) & =\sum_{k=1}^{K}\sum_{j=1}^{n_{k}}(-u_{l,j}(t_{k})\;\text{log}\;(p_{j}(t_{k})b(t_{k}))+p_{j}(t_{k})b(t_{k}))\\ & +(-s_{l,j}(t_{k})\;\text{log}\;(p_{j}(t_{k})c(t_{k}))+p_{j}(t_{k})c(t_{k})). \end{array}$$ (37)

Utilizing (12), we got ∂*b*(*t*)/∂*α* = (1 − *e*^−*βt*^)/*β* and $\partial c(t)/\partial \alpha =(1-e^{-\gamma_{s}t})/\gamma_{s}+(e^{-\gamma_{s}t}-e^{-\beta t})/(\gamma_{s}-\beta )$ when *β* ≠ *γ*_*s*_. The case for *β* = *γ*_*s*_ is similar. So ∂*L*(*α*, *β*_*t*_, *γ*_*s*_)/∂*α* = 0 has a closed form solution

$$\begin{array}{c} \alpha \left(\beta ,\gamma_{s}\right)=\frac{\left\langle u_{l,j}\left(t_{k}\right)+s_{l,j}\left(t_{k}\right)\right\rangle }{\left\langle p_{j}\left(t_{k}\right)\left(\frac{\partial b}{\partial \alpha }\left(t_{k}\right)+\frac{\partial c}{\partial \alpha }\left(t_{k}\right)\right)\right\rangle }. \end{array}$$ (38)

However ∂*L*/∂*β* = 0 and ∂*L*/∂*γ*_*s*_ = 0 have no closed form solution, and we need to solve these equations by iterations. The choice of initial values is similar to the CSP-Baseline case. We took the initial value of *β* and *γ*_*s*_ as the solution from Dynamo [26] under the steady-state assumption, which we denoted as *β*_0_, *γ*_*s*,0_. And then the initial value of *α* is taken as *α*_0_ = *α*(*β*_0_, *γ*_*s*,0_).

In CSP-Switching, we need to maximize the log-likelihood function

$$\begin{array}{cc} \ell (p_{\text{off}}) & =\sum_{k=1}^{K}\sum_{j=1}^{n_{k}}I_{\left\{l_{j}\left(t_{k}\right)=0\right\}}\;\text{log}\left(\text{ZIP}\left(p_{j}a\left(t_{k}\right),p_{\text{off}}\right){|}_{0}\right)\\ & +I_{\left\{l_{j}\left(t_{k}\right)>0\right\}}\;\text{log}\left(\text{ZIP}\left(p_{j}a\left(t_{k}\right),p_{\text{off}}\right){|}_{l_{j}\left(t_{k}\right)}\right), \end{array}$$ (39)

where ZIP(λ, *p*_off_)|_*n*_ ≔ Prob(*X* = *n*) is the probability of *X* = *n* for a ZIP-distributed random variable *X* with parameters λ and *p*_off_. It is equivalent to minimizing the loss function

$$\begin{array}{c} \begin{array}{cc} L(\alpha ,\gamma_{t},p_{\text{off}}) & =\sum_{k=1}^{K}\sum_{j=1}^{n_{k}}-\text{log}\left(\text{ZIP}\left(p_{j}a\left(t_{k}\right),p_{\text{off}}\right){|}_{0}\right)-\\ & I_{\left\{l_{j}\left(t_{k}\right)>0\right\}}(\text{log}\left(1-p_{\text{off}}\right)+l_{j}\left(t_{k}\right)\;\text{log}\left(p_{j}\left(t_{k}\right)a\left(t_{k}\right)\right)-p_{j}\left(t_{k}\right)a\left(t_{k}\right)). \end{array} \end{array}$$ (40)

Similar as before, we chose the initial value of *γ*_*t*_, denoted as *γ*_*t*,0_, based on the steady state assumption, and chose the moment estimator

$$\begin{array}{c} p_{\text{off},0}=1-\frac{{\left\langle l_{j}\left(t_{k}\right)\right\rangle }^{2}\left\langle {\left(p_{j}\left(t_{k}\right)\frac{\partial a}{\partial \alpha }\left(t_{k}\right)\right)}^{2}\right\rangle }{{\left\langle p_{j}\left(t_{k}\right)\frac{\partial a}{\partial \alpha }\left(t_{k}\right)\right\rangle }^{2}\left(\left\langle l_{j}{\left(t_{k}\right)}^{2}\right\rangle -\left\langle l_{j}\left(t_{k}\right)\right\rangle \right)} \end{array}$$ (41)

and

$$\begin{array}{c} \alpha_{0}=\frac{\left\langle l_{j}\left(t_{k}\right)\right\rangle }{\left(1-p_{\text{off},0}\right)\left\langle p_{j}\left(t_{k}\right)\frac{\partial a}{\partial \alpha }\left(t_{k}\right)\right\rangle } \end{array}$$ (42)

as the initial values of *p*_off_ and *α*.

According to the biological meaning of the parameters, we added the constraints 0 < *α* < 10*α*_0_, 0 < *β* < 10*β*_0_, 0 < *γ*_*t*_ < 10*γ*_*t*,0_, 0 < *γ*_*s*_ < 10*γ*_*s*,0_ and 0 < *p*_off_ < 1, and we called the SLSQP optimizer in SciPy to solve the above optimization problem.

### Goodness-of-fit test for the distribution evolution in time

In ordinary least squares (OLS) linear regression, people often use

$$\begin{array}{c} R^{2}≔1-\frac{\text{RSS}}{\text{TSS}}=1-\frac{\sum_{i=1}^{N}{\left(y_{i}-{\hat{y}}_{i}\right)}^{2}}{\sum_{i=1}^{N}{\left(y_{i}-{\bar{y}}_{i}\right)}^{2}} \end{array}$$ (43)

to define the goodness of fit, where *y*_*i*_ is the sample observation, ${\hat{y}}_{i}$ is the model prediction, and ${\bar{y}}_{i}$ is the sample mean. For the generalized linear model (GLM), the *R*^2^ can be defined using the deviance *D* and null deviance *D*_0_ [29],

$$\begin{array}{c} R_{D}^{2}≔1-\frac{D}{D_{0}}=1-\frac{-2(\ell \left(\hat{\beta }\right)-\ell_{s})}{-2(\ell_{0}-\ell_{s})}=1-\frac{\ell \left(\hat{\beta }\right)-\ell_{s}}{\ell_{0}-\ell_{s}}, \end{array}$$ (44)

where $\ell (\hat{\beta })$, *ℓ*_0_ and *ℓ*_*s*_ denotes the log-likelihood function of the model with parameter $\hat{\beta }$, the null model (that is, fitted with only the intercept), and the saturated model (that is, fitted with one parameter per sample), respectively. $R_{D}^{2}$ can be seen as a generalization of *R*^2^, which is equal to *R*^2^ when the model is a least squares linear regression [29]. Finally, to overcome the disadvantage of adding more parameters without reducing $R_{D}^{2}$ (similar to *R*^2^), we used adjusted $R_{D}^{2}$ (denoted as ${\bar{R}}_{D}^{2}$) as the goodness of fit of different models, which is defined as

$$\begin{array}{c} {\bar{R}}_{D}^{2}≔1-\frac{D/d_{D}}{D_{0}/d_{D_{0}}}=1-\frac{\left(\ell \left(\hat{\beta }\right)-\ell_{s}\right)/d_{D}}{\left(\ell_{0}-\ell_{s}\right)/d_{D_{0}}}, \end{array}$$ (45)

where *d*_*D*_ and $d_{D_{0}}$ are the degrees of freedom of *D* and *D*_0_, respectively. To more intuitively explain the usefulness of $R_{D}^{2}$, we named it goodness of fit of model in the mian text instead of using adjusted deviance *R*^2^.

In CSP-Baseline, *ℓ*_*s*_ has the closed form

$$\begin{array}{c} \ell_{s}=\sum_{k=1}^{K}\sum_{j=1}^{n_{k}}l_{j}\left(t_{k}\right)\;\text{log}\;(P\left(l_{j}\left(t_{k}\right)\right){|}_{l_{j}\left(t_{k}\right)}). \end{array}$$ (46)

To calculate *ℓ*_0_, we need to maximize the log-likelihood function

$$\begin{array}{c} \ell \left(a_{0}\right)=\sum_{k=1}^{K}\sum_{j=1}^{n_{k}}\;\text{log}\;(P\left(p_{j}\left(t_{k}\right)a_{0}\right){|}_{l_{j}\left(t_{k}\right)}), \end{array}$$ (47)

where *a*_0_ is the intercept. The problem has a closed form solution *a*_0_ = 〈*l*_*j*_(*t*_*k*_)〉/〈*p*_*j*_(*t*_*k*_)〉. In addition, *d*_*D*_ = *N* − 2 and $d_{D_{0}}=N-1$, where *N* is the number of cells.

In CSP-Splicing, *ℓ*_*s*_ has the closed form

$$\begin{array}{c} \ell_{s}=\sum_{k=1}^{K}\sum_{j=1}^{n_{k}}\;\text{log}\;(P\left(u_{l,j}\left(t_{k}\right)\right){|}_{u_{l,j}\left(t_{k}\right)})+\text{log}\;(P\left(s_{l,j}\left(t_{k}\right)\right){|}_{s_{l,j}\left(t_{k}\right)}) \end{array}$$ (48)

To calculate *ℓ*_0_, we need to maximize the log-likelihood function

$$\begin{array}{c} \ell \left(b_{0},c_{0}\right)=\text{log}\;(P\left(p_{j}\left(t_{k}\right)b_{0}\right){|}_{u_{l,j}\left(t_{k}\right)})+\text{log}\;(P\left(p_{j}\left(t_{k}\right)c_{0}\right){)|}_{s_{l,j}\left(t_{k}\right)}) \end{array}$$ (49)

where *b*_0_ and *c*_0_ are intercepts and have closed form solutions *b*_0_ = 〈*u*_*l*,*j*_(*t*_*k*_)〉/〈*p*_*j*_(*t*_*k*_)〉 and *c*_0_ = 〈*s*_*l*,*j*_(*t*_*k*_)〉/〈*p*_*j*_(*t*_*k*_)〉, respectively. In addition, *d*_*D*_ = 2*N* − 3 and $d_{D_{0}}=2N-2$.

In CSP-Switching, to calculate *ℓ*_*s*_, we need to maximize the log-likelihood function

$$\begin{array}{cc} \ell (\alpha ,\gamma_{t},p_{\text{off}}) & =\sum_{k=1}^{K}\sum_{j=1}^{n_{k}}I_{\left\{l_{j}\left(t_{k}\right)=0\right\}}\;\text{log}\;(\text{ZIP}\left(0,p_{\text{off}}\right){|}_{0})\\ & +I_{\left\{l_{j}\left(t_{k}\right)>0\right\}}\;\text{log}\;(\text{ZIP}\left(l_{j}\left(t_{k}\right),p_{\text{off}}\right){|}_{l_{j}\left(t_{k}\right)})\\ & =\sum_{k=1}^{K}\sum_{j=1}^{n_{k}}I_{\left\{l_{j}\left(t_{k}\right)>0\right\}}\;\text{log}\;(\text{ZIP}\left(l_{j}\left(t_{k}\right),p_{\text{off}}\right){|}_{l_{j}\left(t_{k}\right)}) \end{array}$$ (50)

When *p*_off_ is equal to zero, Eq (50) is maximized, and the closed form solution of *ℓ*_*s*_ is

$$\begin{array}{c} \ell_{s}=\sum_{k=1}^{K}\sum_{j=1}^{n_{k}}I_{\left\{l_{j}\left(t_{k}\right)>0\right\}}(l_{j}\left(t_{k}\right)\;\text{log}\left(l_{j}\left(t_{k}\right)\right)-l_{j}\left(t_{k}\right)-\text{log}\left(l_{i}\left(t_{k}\right)!\right)). \end{array}$$ (51)

To calculate *ℓ*_0_, we need to maximize the log-likelihood function

$$\begin{array}{cc} \ell (a_{0},p_{\text{off}})= & \sum_{k=1}^{K}\sum_{j=1}^{n_{k}}I_{\left\{l_{j}\left(t_{k}\right)=0\right\}}\;\text{log}\left(\text{ZIP}\left(p_{j}a_{0},p_{\text{off}}\right){|}_{0}\right)\\ & +I_{\left\{l_{j}\left(t_{k}\right)>0\right\}}\;\text{log}\left(\text{ZIP}\left(p_{j}a_{0},p_{\text{off}}\right){|}_{l_{j}\left(t_{k}\right)}\right). \end{array}$$ (52)

Similar to solving Eq (40), *p*_off,0_ and *a*_0_ were initialized using moment estimators with additional constraints 0 < *p*_off_ < 1 and 0 < *a* < 10*a*_0_. We then called the SLSQP optimizer in SciPy to solve the problem. In addition, *d*_*D*_ = *N* − 2 and $d_{D_{0}}=N-1$. Before projecting high-dimensional RNA velocities to low dimensions for visualization, we first pick genes with higher goodness-of-fit. The default setting is that the top 40% of genes are picked, and this percentage can be specified by the user.

### Post-processing for cell-specific parameters

In our cell-specific modeling of gene expression, we only assumed that *γ*_*t*_ (in CSP-Baseline and CSP-Switching) and *γ*_*s*_ (in CSP-Splicing) are constants over cells and are inferred based on the corresponding stochastic models, while the other parameters are cell-specific and continuously dependent on gene expressions. This relaxed assumption implies that only the degradation rate is common to all cells, and only cells with similar gene expressions have similar other parameters (due to continuous dependence). To realize this assumption, we first constructed the k-nearest neighbor (kNN) graph of cells by a data preprocessing. The cell-specific parameter inference was performed by applying the inference to the kNN graph for each cell with local constant parameter assumption and already inferred degradation rates. In other deterministic model-based methods to infer RNA velocity (either unspliced/spliced-based or new/total-based) [1, 2, 26], they also construct similar kNN graph and perform kNN smoothing on the data based on this graph. This post-processing step of ours can be seen as the generalization of the usual kNN smoothing to our stochastic setting, as we model discrete counts. The inference details for our three models were shown as below.

In CSP-Baseline, we have

$$\begin{array}{c} l_{i}\left(t_{k}\right)∼\text{Poisson}\left(p_{i}a_{j}\left(t_{k}\right)\right),\;\forall i\in N_{j,t_{k}}, \end{array}$$ (53)

where $N_{j,t_{k}}$ denotes the set of top *k* (default is 30) cells that have the most similar gene expressions as the *j*th cell with labeling duration *t*_*k*_ (including itself) and $a_{j}\left(t_{k}\right)=\alpha_{j}\left(t_{k}\right)\left(1-e^{-\gamma_{t}t_{k}}\right)/\gamma_{t}$. Assuming that *γ*_*t*_ has been inferred, we can obtain a local estimator

$$\begin{array}{c} \frac{\sum_{i\in N_{j,t_{k}}}l_{i}\left(t_{k}\right)}{\sum_{i\in N_{j,t_{k}}}p_{i}\left(t_{k}\right)}=a_{j}\left(t_{k}\right)=\frac{\alpha_{j}\left(t_{k}\right)}{\gamma_{t}}\left(1-e^{-\gamma_{t}t_{k}}\right) \end{array}$$ (54)

by using the MLE. Define ${\hat{l}}_{j}\left(t_{k}\right)=\left(\sum_{i\in N_{j,t_{k}}}l_{i}\left(t_{k}\right)\right)/\left(\sum_{i\in N_{j,t_{k}}}p_{i}\left(t_{k}\right)\right)$. Then the cell-specific transcription rate *α*_*j*_(*t*_*k*_) has a closed form solution

$$\begin{array}{c} \alpha_{j}\left(t_{k}\right)={\hat{l}}_{j}\left(t_{k}\right)\gamma_{t}/\left(1-e^{-\gamma_{t}t_{k}}\right). \end{array}$$ (55)

In CSP-Splicing, we have

$$\begin{array}{c} \left(u_{l,i}\left(t_{k}\right),s_{l,i}\left(t_{k}\right)\right)∼\text{independent}\;\text{Poisson}\left(p_{i}b_{j}\left(t_{k}\right),p_{i}c_{j}\left(t_{k}\right)\right),\;\forall i\in N_{j,t_{k}}. \end{array}$$ (56)

Similarly, assuming *γ*_*s*_ has been inferred, and defining the local estimators

$$\begin{array}{c} {\hat{u}}_{l,j}\left(t_{k}\right)=\frac{\sum_{i\in N_{j,t_{k}}}u_{l,i}\left(t_{k}\right)}{\sum_{i\in N_{j,t_{k}}}p_{i}\left(t_{k}\right)},\;\;{\hat{s}}_{l,j}\left(t_{k}\right)=\frac{\sum_{i\in N_{j,t_{k}}}s_{l,i}\left(t_{k}\right)}{\sum_{i\in N_{j,t_{k}}}p_{i}\left(t_{k}\right)}, \end{array}$$ (57)

we have

$$\begin{array}{cc} {\hat{u}}_{l,j}\left(t_{k}\right) & =b_{j}\left(t_{k}\right)=\frac{\alpha_{j}\left(t_{k}\right)}{\beta_{j}\left(t_{k}\right)}\left(1-e^{-\beta_{j}\left(t_{k}\right)t_{k}}\right),\\ {\hat{s}}_{l,j}\left(t_{k}\right) & =c_{j}\left(t_{k}\right)=\frac{\alpha_{j}\left(t_{k}\right)}{\gamma_{s}}\left(1-e^{-\gamma_{s}t_{k}}\right)+\frac{\alpha_{j}\left(t_{k}\right)}{\gamma_{s}-\beta_{j}\left(t_{k}\right)}\left(e^{-\gamma_{s}t_{k}}-e^{-\beta_{j}\left(t_{k}\right)t_{k}}\right), \end{array}$$ (58)

which is a nonlinear system. We have

$$\begin{array}{c} \frac{{\hat{s}}_{l,j}\left(t_{k}\right)}{{\hat{u}}_{l,j}\left(t_{k}\right)}=\frac{\beta_{j}\left(t_{k}\right)\left(1-e^{-\gamma_{s}t_{k}}\right)}{\gamma_{s}\left(1-e^{-\beta_{j}\left(t_{k}\right)t_{k}}\right)}+\frac{\beta_{j}\left(t_{k}\right)\left(e^{-\gamma_{s}t_{k}}-e^{-\beta_{j}\left(t_{k}\right)t_{k}}\right)}{\left(\gamma_{s}-\beta_{j}\left(t_{k}\right)\right)\left(1-e^{-\beta_{j}\left(t_{k}\right)t_{k}}\right)}. \end{array}$$ (59)

To solve *β*_*j*_(*t*_*k*_), we set its initial value as previously inferred *β* by global constant assumption. We then call the *foot* function in SciPy to solve the nonlinear equation (59) to get *β*_*j*_(*t*_*k*_). The *α*_*j*_(*t*_*k*_) has a closed form solution

$$\begin{array}{c} \alpha_{j}\left(t_{k}\right)={\hat{u}}_{l,j}\left(t_{k}\right)\beta_{j}\left(t_{k}\right)/\left(1-e^{-\beta_{j}\left(t_{k}\right)t_{k}}\right). \end{array}$$ (60)

In summary, in CSP-Splicing, we can infer the cell-specific transcription rate *α*_*j*_(*t*_*k*_) and splicing rate *β*_*j*_(*t*_*k*_).

In CSP-Switching, we have

$$\begin{array}{c} l_{i}\left(t_{k}\right)∼\text{ZIP}\left(p_{i}a_{j}\left(t_{k}\right),p_{\text{off},j}\left(t_{k}\right)\right),\;\forall i\in N_{j,t_{k}}. \end{array}$$ (61)

When computing RNA velocity, we only need to know *α*_*j*_(*t*_*k*_)(1 − *p*_off,*j*_(*t*_*k*_)) as a whole, and not their respective values (see next subsection). To simplify the computation, we used the moment estimation instead of MLE, and got

$$\begin{array}{c} {\hat{l}}_{j}\left(t_{k}\right)=\left(1-p_{\text{off},j}\right)a_{j}\left(t_{k}\right)=\frac{\left(1-p_{\text{off},j}\left(t_{k}\right)\right)\alpha_{j}\left(t_{k}\right)}{\gamma_{t}}\left(1-e^{-\gamma_{t}t_{k}}\right). \end{array}$$ (62)

Similarly, assuming *γ*_*t*_ has been inferred, *α*_*j*_(*t*_*k*_)(1 − *p*_off,*j*_(*t*_*k*_)) has a closed form solution

$$\begin{array}{c} \alpha_{j}\left(t_{k}\right)\left(1-p_{\text{off},j}\left(t_{k}\right)\right)={\hat{l}}_{j}\left(t_{k}\right)\gamma_{t}/\left(1-e^{-\gamma_{t}t_{k}}\right). \end{array}$$ (63)

### Reduction from stochastic to deterministic models for RNA velocity

We used discrete counts data in the proposed parameter inference and goodness-of-fit calculation via stochastic models. However, when we need to compute and visualize the RNA velocity, we should take the reduction from stochastic to deterministic models to get the mean velocity. Below we would show the reduction process and reveal the connection between the stochastic and their corresponding deterministic models.

In CSP-Baseline, let us denote the mean value of $\tilde{l}\left(t\right)$ by $\langle \tilde{l}\left(t\right)\rangle$, which is defined as $\langle \tilde{l}\left(t\right)\rangle =\sum_{n=1}^{\infty }n{\tilde{P}}_{n}\left(t\right)$. From Eq (5) we can obtain the deterministic equation after suitable algebraic manipulations

$$\begin{array}{cc} \frac{d\langle \tilde{l}\left(t\right)\rangle }{dt} & =\sum_{n=1}^{\infty }n\frac{d{\tilde{P}}_{n}\left(t\right)}{dt}\\ & =\sum_{n=1}^{\infty }n\left(-\left(\alpha +n\gamma_{t}\right){\tilde{P}}_{n}+\alpha {\tilde{P}}_{n-1}+\gamma_{t}\left(n+1\right){\tilde{P}}_{n+1}\right)\\ & =\alpha -\gamma_{t}\left\langle \tilde{l}\left(t\right)\right\rangle . \end{array}$$ (64)

Similarly, the mean value of total RNA $\tilde{r}\left(t\right)$ satisfies the equation

$$\begin{array}{c} \frac{d\langle \tilde{r}\left(t\right)\rangle }{dt}=\alpha -\gamma_{t}\left\langle \tilde{r}\left(t\right)\right\rangle . \end{array}$$ (65)

Since the initial value of $\tilde{l}\left(t\right)$ is zero, we got

$$\begin{array}{c} \left\langle \tilde{l}\left(t\right)\right\rangle =a\left(t\right)=\frac{\alpha }{\gamma_{t}}\left(1-e^{-\gamma_{t}t}\right). \end{array}$$ (66)

In CSP-Splicing, the marginal PMFs of ${\tilde{u}}_{l}\left(t\right)$ and ${\tilde{s}}_{l}\left(t\right)$ are

$$\begin{array}{cc} {\tilde{P}}_{m,\cdot }\left(t\right) & ≔\text{Prob}({\tilde{u}}_{l}\left(t\right)=m)=\sum_{n=0}^{\infty }{\tilde{P}}_{m,n}\left(t\right),\\ {\tilde{P}}_{\cdot ,n}\left(t\right) & ≔\text{Prob}({\tilde{s}}_{l}\left(t\right)=n)=\sum_{m=0}^{\infty }{\tilde{P}}_{m,n}\left(t\right), \end{array}$$ (67)

respectively. The mean values of ${\tilde{u}}_{l}\left(t\right)$ and ${\tilde{s}}_{l}\left(t\right)$ have the form $\langle {\tilde{u}}_{l}\left(t\right)\rangle =\sum_{m=1}^{\infty }m{\tilde{P}}_{m,\cdot }\left(t\right)$ and $\langle {\tilde{s}}_{l}\left(t\right)\rangle =\sum_{n=1}^{\infty }n{\tilde{P}}_{\cdot ,n}\left(t\right)$. From the CME (10), we can obtain

$$\begin{array}{cc} \frac{d\langle {\tilde{u}}_{l}\left(t\right)\rangle }{dt} & =\sum_{m=1}^{\infty }m\partial_{t}{\tilde{P}}_{m,\cdot }\left(t\right)=\sum_{m=1}^{\infty }m\sum_{n=0}^{\infty }\partial_{t}{\tilde{P}}_{m,n}\left(t\right)\\ & =\sum_{m=1}^{\infty }m\sum_{n=0}^{\infty }\alpha \left({\tilde{P}}_{m-1,n}-{\tilde{P}}_{mn}\right)+\beta \left(\left(m+1\right){\tilde{P}}_{m+1,n-1}-m{\tilde{P}}_{mn}\right)\\ & +\gamma_{s}\left(\left(n+1\right){\tilde{P}}_{m,n+1}-n{\tilde{P}}_{mn}\right)\\ & =\alpha -\beta \langle {\tilde{u}}_{l}\left(t\right)\rangle , \end{array}$$ (68)

and

$$\begin{array}{cc} \frac{d\langle {\tilde{s}}_{l}\left(t\right)\rangle }{dt} & =\sum_{n=1}^{\infty }n\partial_{t}{\tilde{P}}_{\cdot ,n}\left(t\right)=\sum_{n=1}^{\infty }n\sum_{m=0}^{\infty }\partial_{t}{\tilde{P}}_{m,n}\left(t\right)\\ & =\sum_{n=1}^{\infty }n\sum_{m=0}^{\infty }\alpha \left({\tilde{P}}_{m-1,n}-{\tilde{P}}_{mn}\right)+\beta \left(\left(m+1\right){\tilde{P}}_{m+1,n-1}-m{\tilde{P}}_{mn}\right)\\ & +\gamma_{s}\left(\left(n+1\right){\tilde{P}}_{m,n+1}-n{\tilde{P}}_{mn}\right)\\ & =\beta \left\langle {\tilde{u}}_{l}\left(t\right)\right\rangle -\gamma_{s}\left\langle {\tilde{s}}_{l}\left(t\right)\right\rangle . \end{array}$$ (69)

Similarly, we can derive the equations for the mean values of total unspliced and spliced mRNA $\left(\tilde{u}\left(t\right),\tilde{s}\left(t\right)\right)$:

$$\begin{array}{c} \frac{d\langle \tilde{u}\left(t\right)\rangle }{dt}=\alpha -\beta \langle \tilde{u}\left(t\right)\rangle ,\\ \frac{d\langle \tilde{s}\left(t\right)\rangle }{dt}=\beta \left\langle \tilde{u}\left(t\right)\right\rangle -\gamma_{s}\left\langle \tilde{s}\left(t\right)\right\rangle . \end{array}$$ (70)

Since the initial value of $\left({\tilde{u}}_{l}\left(t\right),{\tilde{s}}_{l}\left(t\right)\right)$ is (0, 0), we got

$$\begin{array}{c} \left\langle {\tilde{u}}_{l}\left(t\right)\right\rangle =b\left(t\right)=\frac{\alpha }{\beta }\left(1-e^{-\beta t}\right) \end{array}$$ (71)

and

$$\begin{array}{c} \begin{array}{c} \left\langle {\tilde{s}}_{l}\left(t\right)\right\rangle =c\left(t\right)=\{\begin{array}{cc} \frac{\alpha }{\gamma_{s}}\left(1-e^{-\gamma_{s}t}\right)+\frac{\alpha }{\gamma_{s}-\beta }\left(e^{-\gamma_{s}t}-e^{-\beta t}\right), & \beta \neq \gamma_{s},\\ \frac{\alpha }{\beta }\left(1-e^{-\beta t}\right)-\alpha te^{-\beta t}, & \beta =\gamma_{s}. \end{array} \end{array} \end{array}$$ (72)

Similar to CSP-Baseline, in CSP-Switching, $d\langle \tilde{l}\left(t\right)\rangle /dt$ and $d\langle \tilde{r}\left(t\right)\rangle /dt$ satisfy the equations

$$\begin{array}{c} \frac{d\langle \tilde{l}\left(t\right)\rangle }{dt}=\left(1-p_{\text{off}}\right)\alpha -\gamma_{t}\left\langle \tilde{l}\left(t\right)\right\rangle ,\\ \frac{d\langle \tilde{r}\left(t\right)\rangle }{dt}=\left(1-p_{\text{off}}\right)\alpha -\gamma_{t}\left\langle \tilde{r}\left(t\right)\right\rangle . \end{array}$$ (73)

Since the initial value of $\tilde{l}\left(t\right)$ is zero, we got

$$\begin{array}{c} \left\langle \tilde{l}\left(t\right)\right\rangle =\frac{(1-p_{\text{off}})\alpha }{\gamma_{t}}\left(1-e^{-\gamma_{t}t}\right). \end{array}$$ (74)

### Computation of RNA velocity

To ease the notation, we denoted the new mRNA after data preprocessing by $\bar{l}\left(t\right)$, defined as

$${\bar{l}}_{j}\left(t_{k}\right)=\frac{1}{|N_{j,t_{k}}|}\sum_{i\in N_{j,t_{k}}}\frac{l_{i}\left(t_{k}\right)}{p_{i}\left(t_{k}\right)},$$

which is different from the true expression $\tilde{l}\left(t\right)$, the discrete counts data *l*(*t*), and the notation $\hat{l}\left(t\right)$ in the post-processing subsection. We would also use the notation $\bar{u}\left(t\right)$, $\bar{s}\left(t\right)$ and $\bar{r}\left(t\right)$ with similar definition.

In CSP-Baseline, only the total RNA velocity can be obtained due to the lack of the splicing stage. From Eq (65), we have

$$\begin{array}{c} v_{\text{total},r_{j}\left(t_{k}\right)}=\alpha_{j}\left(t_{k}\right)-\gamma_{t}{\bar{r}}_{j}\left(t_{k}\right), \end{array}$$ (75)

where ${\bar{r}}_{j}\left(t_{k}\right)$ is the number of total mRNA molecules of the *j*th cell labeled with length *t*_*k*_ after data preprocessing.

In CSP-Splicing, we add the two equations in Eq (70) to obtain

$$\begin{array}{c} \frac{d\langle \tilde{r}\left(t\right)\rangle }{dt}=\frac{d\langle \tilde{u}\left(t\right)\rangle }{dt}+\frac{d\langle \tilde{s}\left(t\right)\rangle }{dt}=\alpha -\gamma_{s}\left\langle \tilde{s}\left(t\right)\right\rangle , \end{array}$$ (76)

and thus get the equation for total RNA velocity

$$\begin{array}{c} v_{\text{total},r_{j}\left(t_{k}\right)}=\alpha_{j}\left(t_{k}\right)-\gamma_{s}{\bar{s}}_{j}\left(t_{k}\right). \end{array}$$ (77)

In addition, in CSP-Splicing, we can also calculate the spliced RNA velocity by the following equation

$$\begin{array}{c} v_{\text{spliced},s_{j}\left(t_{k}\right)}=\beta_{j}\left(t_{k}\right){\bar{u}}_{j}\left(t_{k}\right)-\gamma_{s}{\bar{s}}_{j}\left(t_{k}\right). \end{array}$$ (78)

Similar to CSP-Baseline, the total RNA velocity in CSP-Switching can be obtained by the equation

$$\begin{array}{c} v_{\text{total},r_{j}\left(t_{k}\right)}=\left(1-p_{\text{off},j}\left(t_{k}\right)\right)\alpha_{j}\left(t_{k}\right)-\gamma_{t}{\bar{r}}_{j}\left(t_{k}\right). \end{array}$$ (79)

### Relationship between *γ*_*t*_ and *γ*_*s*_ and its implications

The difference between Eqs (65) and (76) implies the difference between the total mRNA degradation rate *γ*_*t*_ and spliced mRNA degradation rate *γ*_*s*_. After suitable manipulations, we had the relation between *γ*_*t*_ and *γ*_*s*_ as below

$$\begin{array}{c} \frac{\gamma_{s}}{\gamma_{t}}=\frac{\left\langle \tilde{r}\left(t\right)\right\rangle }{\left\langle \tilde{s}\left(t\right)\right\rangle }. \end{array}$$ (80)

Therefore, we naturally got a method to infer *γ*_*t*_ when *γ*_*s*_ is known. Specifically, we first performed a zero-intercept linear regression

$$\begin{array}{c} {\bar{r}}_{j}\left(t_{k}\right)=k{\bar{s}}_{j}\left(t_{k}\right) \end{array}$$ (81)

to get the slope *k*. Then we computed *γ*_*t*_ by *γ*_*t*_ = *γ*_*s*_/*k*. Therefore, we can also infer *γ*_*t*_ and compute the total RNA velocity by Eq (75) in CSP-Splicing.

We would also like to point out that CSP-Baseline and CSP-Splicing are incompatible upon assuming that *γ*_*t*_ and *γ*_*s*_ are both constants. These two assumptions usually do not hold simultaneously. Otherwise, from Eq (80) we knew that $\langle \tilde{s}\left(t\right)\rangle /\langle \tilde{r}\left(t\right)\rangle$ is a constant, which is equivalent to that $\langle \tilde{u}\left(t\right)\rangle /\langle \tilde{r}\left(t\right)\rangle$ is a constant, i.e., $\gamma_{t}(1-e^{-\beta t})/(\beta (1-e^{-\gamma_{t}t}))$ is a constant. But this is only true when *β* and *γ*_*t*_ are equal.

### Robust analysis of the parameter inference in the CSP-Baseline

When *γ*_*t*_*t* is small, $1-e^{-\gamma_{t}t}\sim \gamma_{t}t$ holds, then

$$\begin{array}{c} l\left(t\right)=\frac{\alpha (1-e^{-\gamma_{t}t})}{\gamma_{t}}\approx \alpha t, \end{array}$$ (82)

which implies that from the mean perspective the nonlinear fitting of *α* and *γ*_*t*_ degenerated into a linear fitting of *α* at this point. For a more precise analysis, let $\partial a(t)/\partial \alpha =(1-e^{-\gamma_{t}t})/\gamma_{t}$, we have ∂*ℓ*(*α*, *γ*_*t*_)/∂*α* = 0 is equivalent to

$$\begin{array}{c} \alpha \left(\gamma_{t}\right)=\frac{\sum_{k=1}^{K}\sum_{j=1}^{n_{k}}l_{j}\left(t_{k}\right)}{\sum_{k=1}^{K}\sum_{j=1}^{n_{k}}p_{j}\left(t_{k}\right)\partial a\left(t_{k}\right)/\partial \alpha }. \end{array}$$ (83)

But when $1-e^{-\gamma_{t}t}\sim \gamma_{t}t$ holds, ∂*a*(*t*)/∂*α* ≈ *t*, then we have

$$\begin{array}{c} \alpha \approx \frac{\sum_{k=1}^{K}\sum_{j=1}^{n_{k}}l_{j}\left(t_{k}\right)}{\sum_{k=1}^{K}\sum_{j=1}^{n_{k}}p_{j}\left(t_{k}\right)t_{k}} \end{array}$$ (84)

is a constant, which we denoted by *α*_cons_. In addition, to quantitatively measure the robustness of inference on *γ*_*t*_, since the optimal parameter is always located where the gradient is zero, we defined the *l*_1_-norm of the derivative of the loss function with respect to *γ*_*t*_ restricted to ∂*ℓ*/∂*α* = 0 (i.e. black line),

$$\begin{array}{c} {\left\|\frac{d\ell }{d\gamma_{t}}{|}_{\frac{\partial \ell }{\partial \alpha }=0}\left(\gamma_{t}\right)\right\|}_{l_{1}}=\int_{0}^{\gamma_{t,\text{max}}}|\frac{d\ell }{d\gamma_{t}}|d\gamma_{t}, \end{array}$$ (85)

as a measure of robustness. Since the half-life of the total mRNA molecules is usually not less than half an hour, we took *γ*_*t*,max_ = 1.5.

### Definition of correctness and consistency of velocity

The correctness of cell velocities is defined as follows: Consider the cell *i* with position *x*_*i*_ and velocity *v*_*i*_. Define its one-step extrapolated position as *x*_*i*_ + *v*_*i*_. We say that *v*_*i*_ is correct (correctness index = 1) if the cell *j* closest to the extrapolated position *x*_*i*_ + *v*_*i*_ ranks after *i* in the temporal ordering. Otherwise the correctness does not hold and we set the correctness index to be 0. Thus the average correctness refers to the percentage of correct velocities. Because the boundaries of the cell cycle being estimated are not clear and sharp, we did not use the RNA velocity benchmark metric cross-boundary direction (CBDir) proposed by Qiao et al. [12] and widely used for comparison of RNA velocity methods.

The consistency means the extent to which the velocity of one cell is consistent with the velocities of its neighboring cells, and we use the average correlation coefficient proposed in scVelo [2] to measure this consistency.

### Calculation of the duration of each cell cycle phase

After the total RNA velocities are obtained, we can evaluate the time of each phase of a cell cycle based on them. Specifically, we first pick *k* cells $x_{i}^{0}$ (*i* = 1, 2, …, *k*) whose relative positions are closest to 0 as a cell group, calculate their average expression ${\bar{x}}^{0}$ and velocity ${\bar{v}}^{0}$ as the initial expression *x*^0^ and velocity *v*^0^, and extrapolate the state of the cell group with a short time step *dt*, that is, *x*^1^ = *x*^0^ + *v*^0^*dt*. We then search for another *k* cells $x_{i}^{1}$ (*i* = 1, 2, …, *k*) which are closest to the extrapolated state *x*^1^, set the majority of the phase of these *k* cells to the phase of *x*^1^, and set their average velocity ${\bar{v}}^{1}$ as *v*^1^ for the second cell group. Next, the extrapolation and local *k*-cells group identification step can be repeated until a given threshold of the relative position is exceeded. In the actual calculation, we set *k* = 300, *dt* = 0.01, and the threshold of the relative position to be 88% quantile of all relative positions. The above approach for processing the cell groups instead of cells themselves is to reduce the data noise by local averaging.

### Generation of simulation data

In this paper we generate two simulation datasets, one of which is bifurcated one-shot dataset following VeloSim’s [31] flow, and the other is a non-steady-state kinetics dataset following scVelo’s [2] model.

The bifurcated one-shot dataset is generated as described below. First we set the maximum observation time to *T* and the labeling time *t*_*l*_. The observation times for half of the cells were generated randomly with a uniform distribution [*t*_*l*_, *T*/2], and the other half of the cells were divided into two equal parts, which were generated randomly with a uniform distribution [*T*/2, *T*]. We then followed SymSim [30] and VeloSim [31] to generate cell extrinsic variability factor(EVF)s and gene effect vector and used theme to generate the cell-gene-wise transcription rate *α*. Splicing rate *β* and degradation rate *γ*_*s*_ are gene-wise and were generated from uniform distributions of [0, 0.5] and [0, 5], respectively. After all kinetic parameters were generated, we used the Gillespie algorithm to generate raw uu, ul, su and sl RNA counts data. We did not consider technical noise when generating the simulated data and therefore set the size factor of all cells to 1.

The non-steady-state kinetics dataset is based on the model in scVelo [2], but ignores the splicing process. First we set the maximum observation time *T* and the *K* labeling times *t*_1_, *t*_2_ ⋯ *t*_*K*_. The observation times for cells are randomly generated with a uniform distribution [*t*_*K*_, *T*], and the labeling times of the cells were randomly selected with equal probability from *t*_1_ to *t*_*K*_. Transcription rate *α* and degradation rate *γ*_*t*_, both of which are gene-wise, were randomly generated with a uniform distribution [0.5, 1] and [0, 0.5], respectively. To generate non-steady-state data, we set the switching time *t*_*s*_ = 0.5*ρ*/*γ*_*t*_, where *ρ* is a random number generated with a uniform distribution [0, 1]. Finally similarly we use Gillespie algorithm to generate raw new and total RNA counts data and do not consider technical noise.

## Supporting information

S1 Fig Stochastic model combined with steady-state assumptions for one-shot experiments, realated to Fig 2. Storm in this figure refers to the inference strategy of CSP-Baseline model combined with the steady state assumption. **A.** Cell quiver plot in the PCA space of the scSLAM-seq dataset [17]. **B.** Degradation rates *γ*_*t*_ estimated with steady-based method in Storm compared to that of the Dynamo method in the scSLAM-seq dataset [17]. **C.** Same as **B**, but for the datasets from the sci-fate [19]. **D.** Same as **B**, but for the datasets from the PerturbSci-Kinetics [22].
(PNG)

S2 Fig Storm analyzes one-shot data with both splicing and labeling without steady-state assumption, realated to Fig 3. **A.** Streamline projected in the PCA space plots of one-shot bifurcation simulation data of cellDancer. **B.** Streamline plot in the UMAP space of the murine intestinal organoid system dataset from scEU-seq [21] of cellDancer. **C.** Heat map of absolute error between estimated and true gene-cell-wise transcription rates *α* of one-shot bifurcation simulation data of Dynamo.
(PNG)

S3 Fig GO (gene ontology) pathway enrichment results of genes with high *α* and *p*_off_ (top 50%) and low *γ*_*t*_, *β*, *α* and *p*_off_ (bottom 50%) in well-fit genes (top 40% of goodness of fit), related to Fig 5 in main text. (PNG)

S4 Fig RNA velocity analysis of the cell cycle dataset, related to Fig 6. The inference strategy involved in this figure is for kinetics/pulse data. **A.** Comparison of spliced RNA velocity streamline visualizations between CSP-Splicing method and Dynamo. **B.** Comparison of the average correctness of spliced velocity in gene expression space RFP_GFP space. The p-values are given by the one-sided Wilcoxon test. **C.** Similar to **B**, but for velocity consistency. **D.** Total RNA velocity streamlines calculated using gene-wise parameters (instead of using gene-cell-wise parameters except for the degradation rate). **Left**: ICSP. **Right**: CSZIP **E.** Comparison of total RNA velocity in *DCBLD2* between CSP-Splicing and CSP-Switching. **F.** Phase portraits of new-total RNA planes of *DCBLD2* of CSP-Splicing and CSP-Switching. Quivers correspond to the total (x-component) or new (y-component) RNA velocity calculated by the different methods. **G.** Similar to **E**, but for gene *HIPK2* of three stochastic methods and Dynamo. **H.** Similar to **F**, but for gene *HIPK2* of three stochastic methods. **I.** The smoothed expression pattern of *HIPK2* across cells.
(PNG)

S5 Fig RNA velocity analysis of the simulated pulse dataset, related to Fig 6. Storm in this figure refers to the inference strategy of CSP-Baseline model for pulse data. **A.** Comparison of total RNA velocity streamline visualizations between Storm and Dynamo in simulated pulse dataset. **B.** Comparison of the estimated degradation rate with the true degradation rate in simulated pulse data. **C.** Distribution plot of the difference between the estimated degradation rate and the true value, including Storm, well-fitted genes in Storm, Dynamo and well-fitted genes in Dynamo.
(PNG)

S6 Fig PAGA analysis of different datasets and different methods. **A.** Comparison of PAGA velocity graph on the neuronal activity under KCl polarization datasets from scNT-seq. Left: Storm; Right: Dynamo. **B.** Comparison of PAGA velocity graph on the cellcycle dataset from scEU-seq. From left to right, from top to bottom, Storm’s CSP-Baseline stochastic model without steady-state assumption, CSP-Baseline stochastic model with steady-state assumption, Dynamo’s deterministic model with steady-state assumption and random velocity. Type annotations were derived from an equal number division of cells into 8 classes based on the relative positions of cells provided by the original scEU-seq study, and the first and last classes were combined into 1 class.
(PNG)
