# Supplementary figures and images for: Storm: Incorporating transient stochastic dynamics to infer the RNA velocity with metabolic labeling information
Source: PLoS Comput Biol. 2024 Nov 18;20(11):e1012606. doi: 10.1371/journal.pcbi.1012606 (PMC11611270; doi:10.1371/journal.pcbi.1012606)

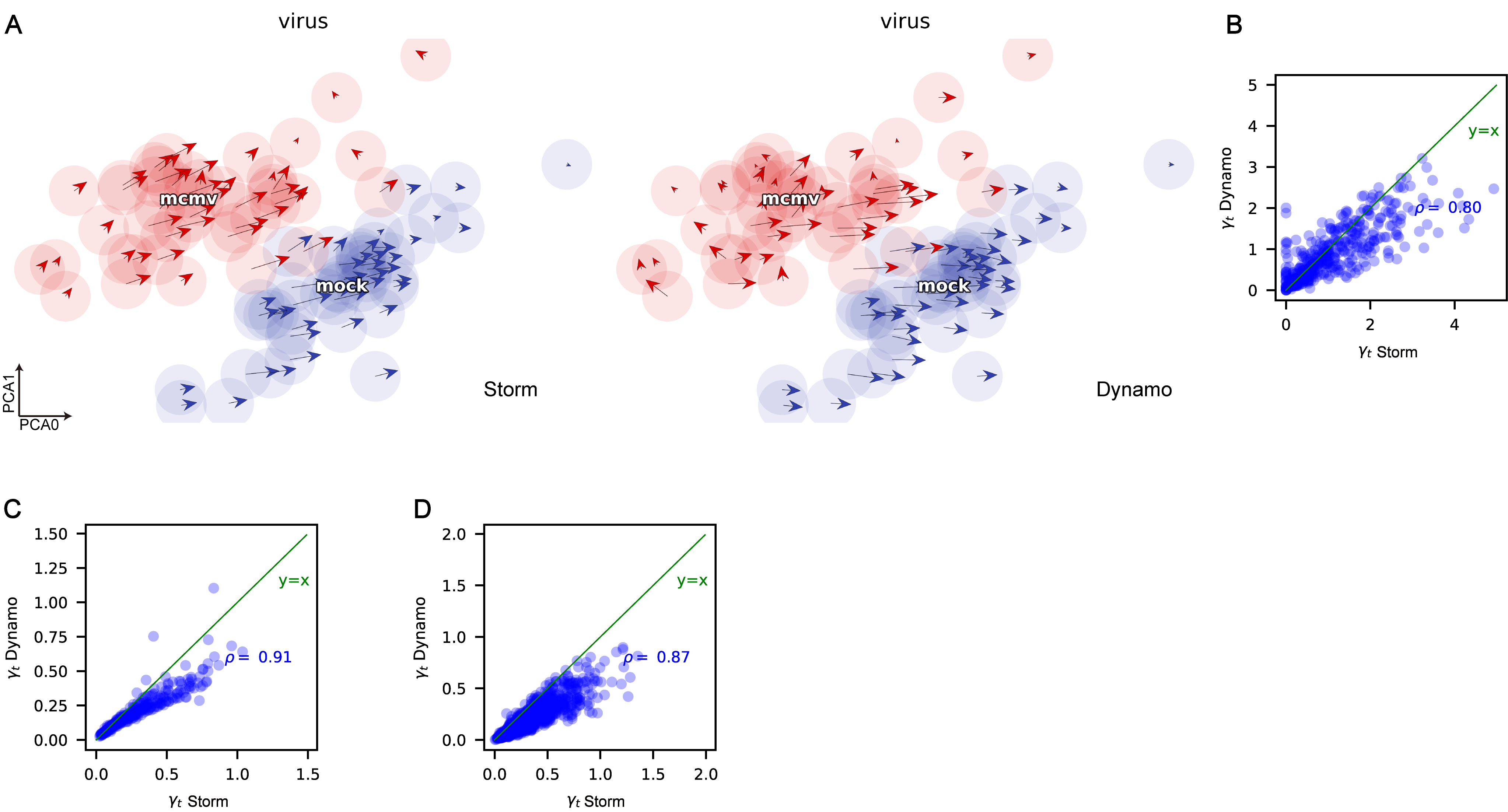

Supplement: S1 Fig — Storm in this figure refers to the inference strategy of CSP-Baseline model combined with the steady state assumption. A. Cell quiver plot in the PCA space of the scSLAM-seq dataset [17]. B. Degradation rates γt estimated with steady-based method in Storm compared to that of the Dynamo method in the scSLAM-seq dataset [17]. C. Same as B, but for the datasets from the sci-fate [19]. D. Same as B, but for the datasets from the PerturbSci-Kinetics [22]. (PNG) [file pcbi.1012606.s001.png]

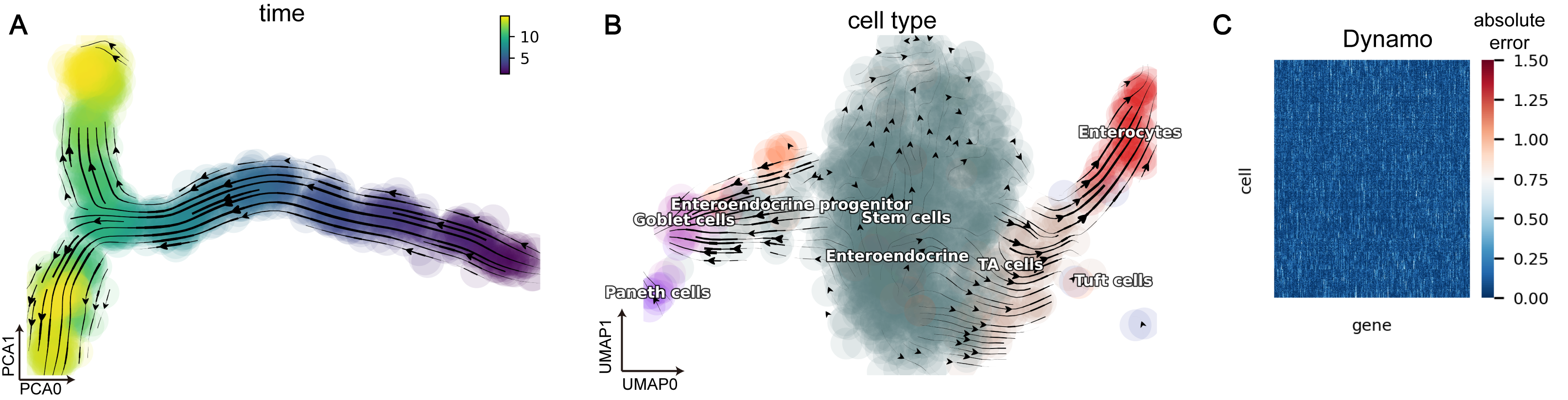

Supplement: S2 Fig — A. Streamline projected in the PCA space plots of one-shot bifurcation simulation data of cellDancer. B. Streamline plot in the UMAP space of the murine intestinal organoid system dataset from scEU-seq [21] of cellDancer. C. Heat map of absolute error between estimated and true gene-cell-wise transcription rates α of one-shot bifurcation simulation data of Dynamo. (PNG) [file pcbi.1012606.s002.png]

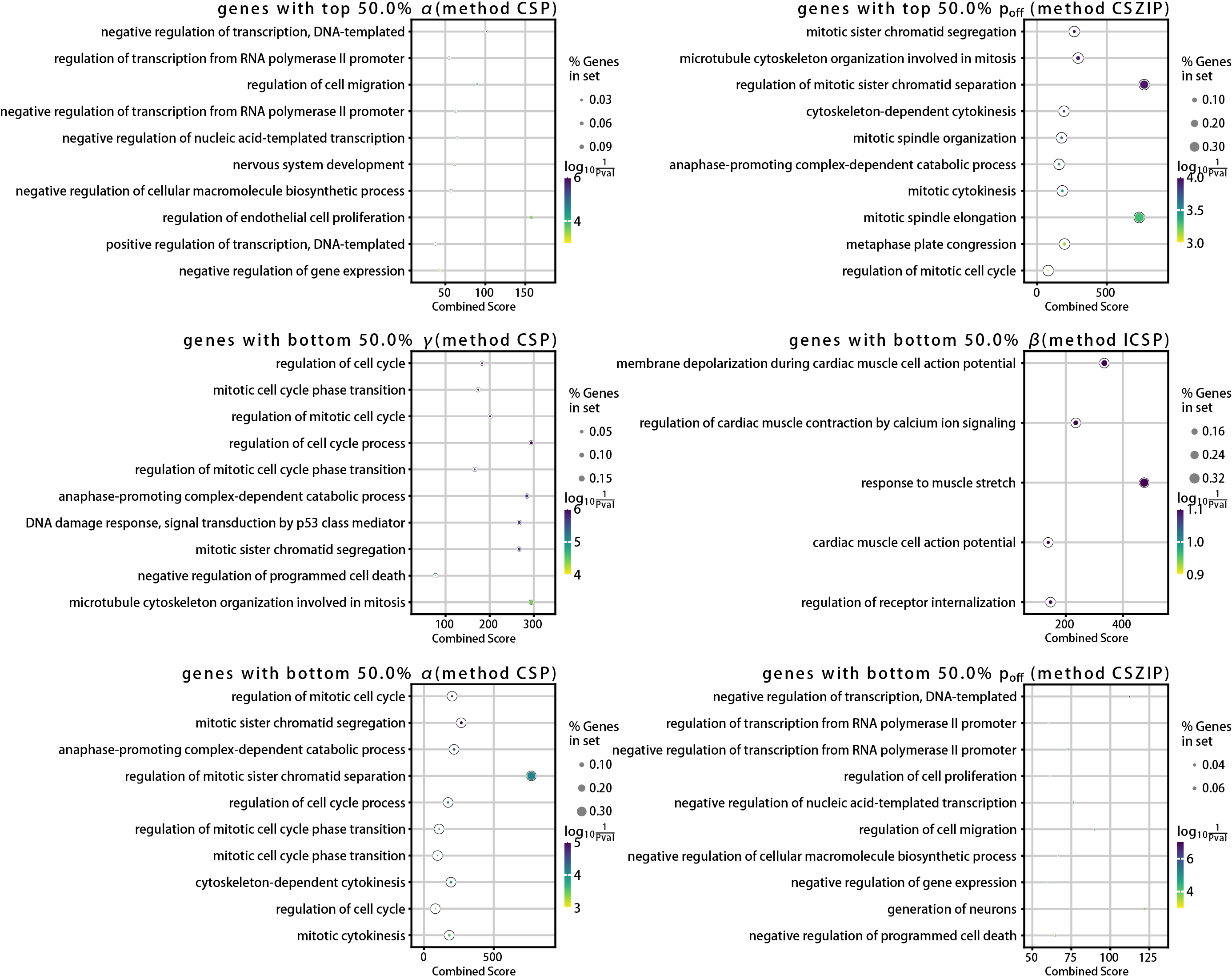

Supplement: S3 Fig — (PNG) [file pcbi.1012606.s003.png]

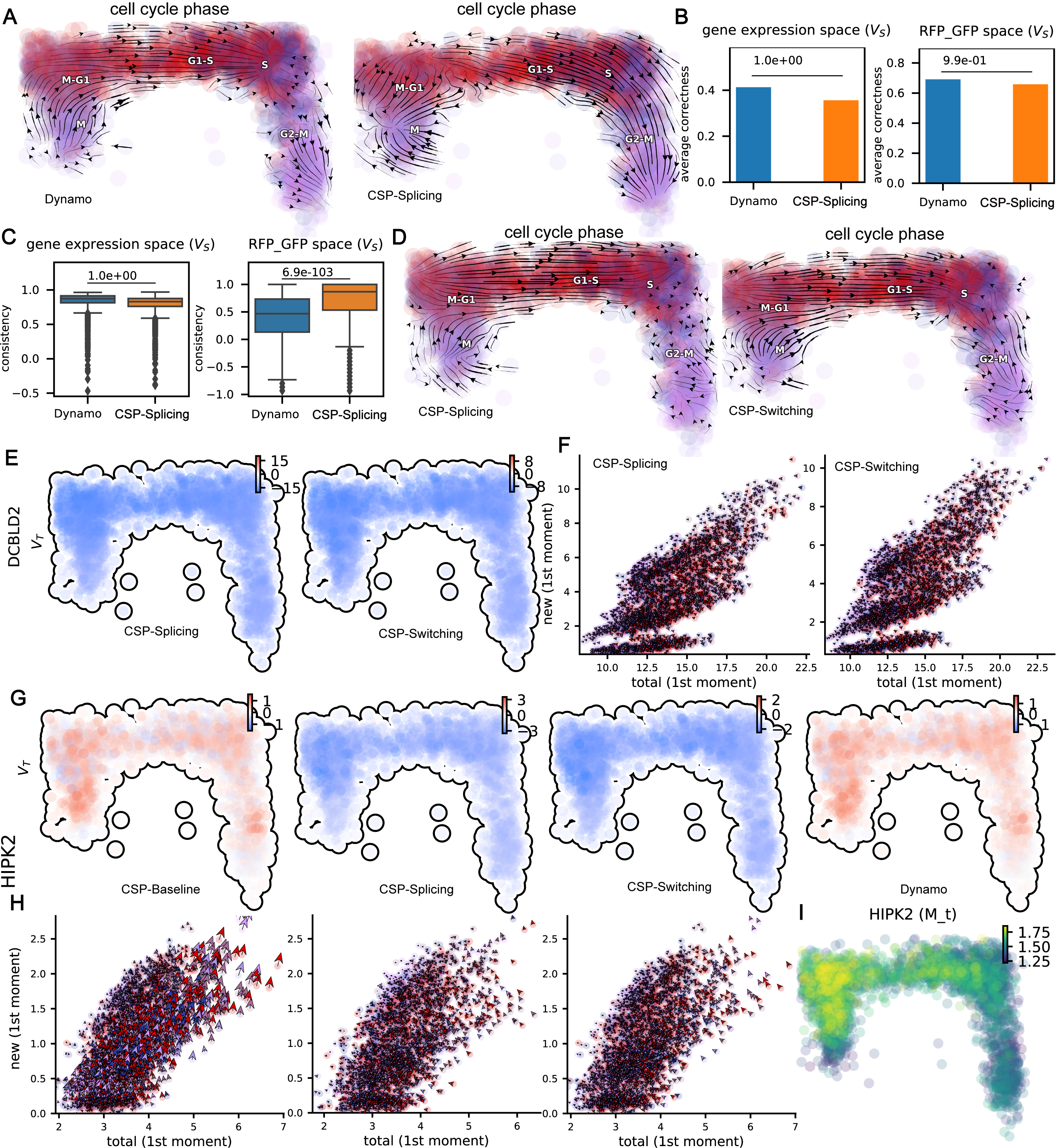

Supplement: S4 Fig — The inference strategy involved in this figure is for kinetics/pulse data. A. Comparison of spliced RNA velocity streamline visualizations between CSP-Splicing method and Dynamo. B. Comparison of the average correctness of spliced velocity in gene expression space RFP_GFP space. The p-values are given by the one-sided Wilcoxon test. C. Similar to B, but for velocity consistency. D. Total RNA velocity streamlines calculated using gene-wise parameters (instead of using gene-cell-wise parameters except for the degradation rate). Left: ICSP. Right: CSZIP E. Comparison of total RNA velocity in DCBLD2 between CSP-Splicing and CSP-Switching. F. Phase portraits of new-total RNA planes of DCBLD2 of CSP-Splicing and CSP-Switching. Quivers correspond to the total (x-component) or new (y-component) RNA velocity calculated by the different methods. G. Similar to E, but for gene HIPK2 of three stochastic methods and Dynamo. H. Similar to F, but for gene HIPK2 of three stochastic methods. I. The smoothed expression pattern of HIPK2 across cells. (PNG) [file pcbi.1012606.s004.png]

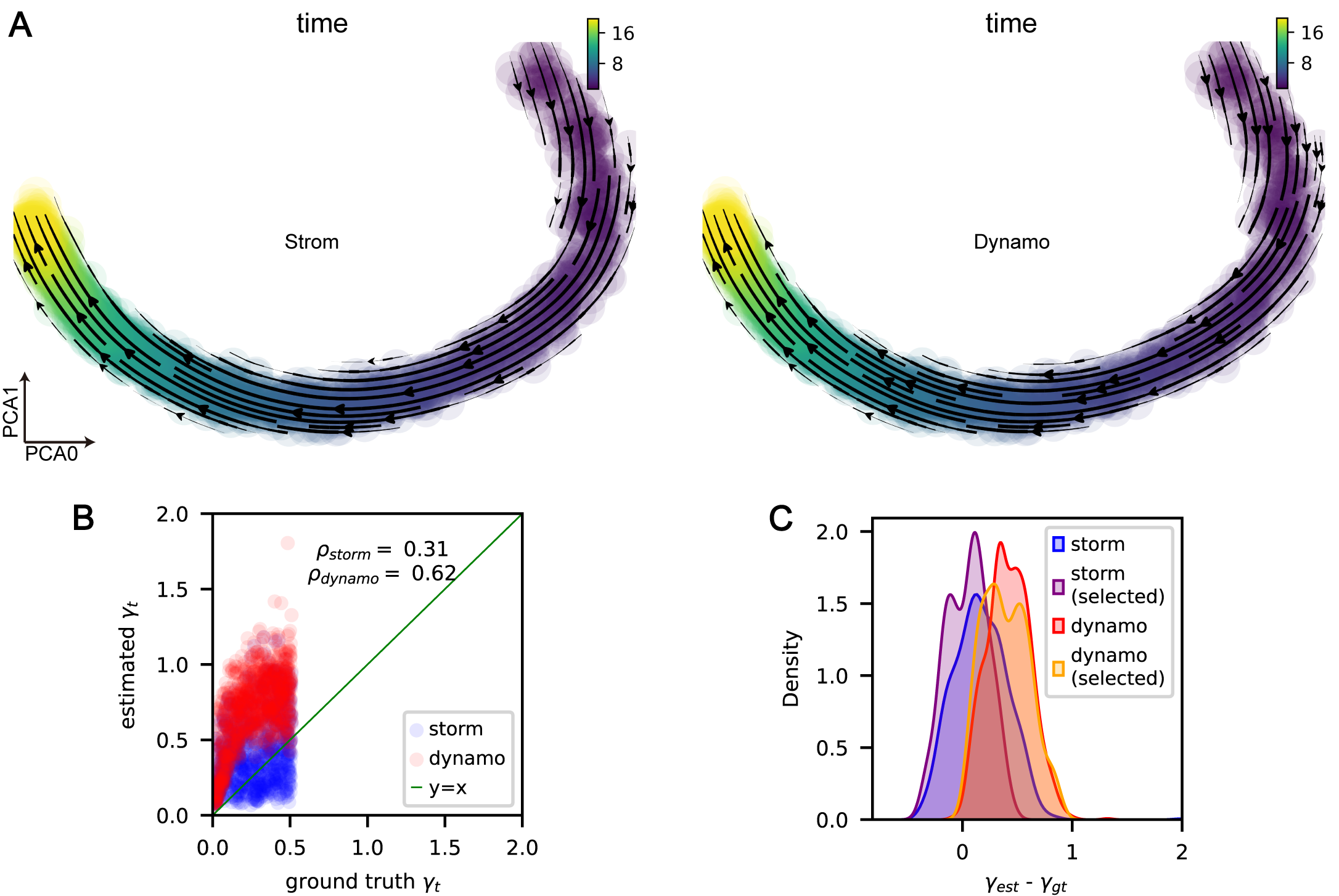

Supplement: S5 Fig — Storm in this figure refers to the inference strategy of CSP-Baseline model for pulse data. A. Comparison of total RNA velocity streamline visualizations between Storm and Dynamo in simulated pulse dataset. B. Comparison of the estimated degradation rate with the true degradation rate in simulated pulse data. C. Distribution plot of the difference between the estimated degradation rate and the true value, including Storm, well-fitted genes in Storm, Dynamo and well-fitted genes in Dynamo. (PNG) [file pcbi.1012606.s005.png]

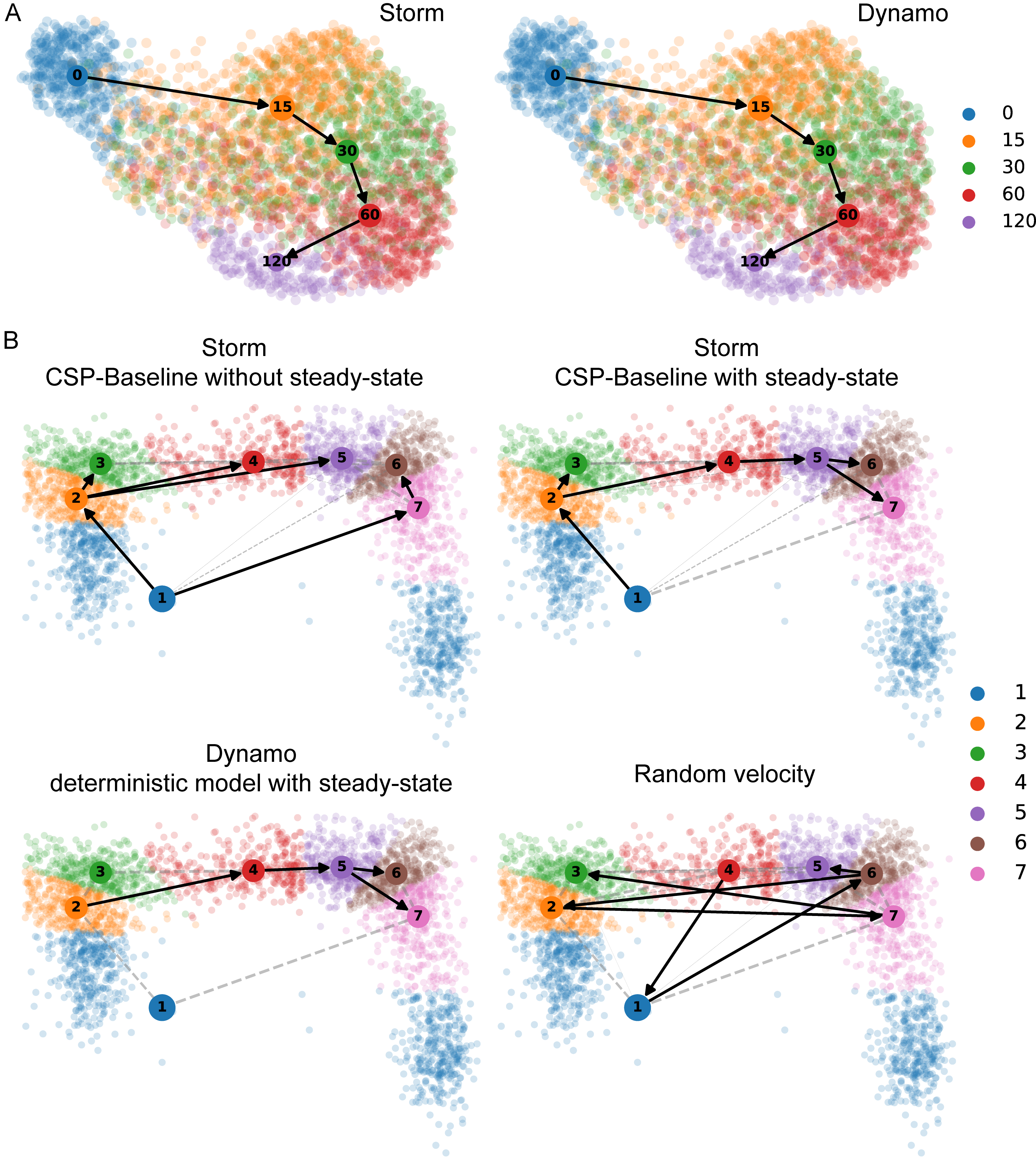

Supplement: S6 Fig — A. Comparison of PAGA velocity graph on the neuronal activity under KCl polarization datasets from scNT-seq. Left: Storm; Right: Dynamo. B. Comparison of PAGA velocity graph on the cellcycle dataset from scEU-seq. From left to right, from top to bottom, Storm’s CSP-Baseline stochastic model without steady-state assumption, CSP-Baseline stochastic model with steady-state assumption, Dynamo’s deterministic model with steady-state assumption and random velocity. Type annotations were derived from an equal number division of cells into 8 classes based on the relative positions of cells provided by the original scEU-seq study, and the first and last classes were combined into 1 class. (PNG) [file pcbi.1012606.s006.png]
